# Solvation-mediated electrostatic shielding of Li anodes by Na^+^ for durable lithium–oxygen batteries

**DOI:** 10.1039/d6sc06234b

**Published:** 2026-10-27

**Authors:** Zhuo Yang, Yichao Cai, Shuo Xu, Yong Lu, Jun Chen

**Affiliations:** a Key Laboratory of Advanced Energy Materials Chemistry (Ministry of Education), State Key Laboratory of Advanced Chemical Power Sources, College of Chemistry, Nankai University Tianjin 300071 China chenabc@nankai.edu.cn

## Abstract

Aprotic lithium–oxygen (Li–O_2_) batteries represent a promising next-generation energy storage technology due to their high theoretical energy density. However, their practical application is severely hampered by lithium dendrite growth and anode failure. Here, we establish a solvation-mediated electrostatic-shielding strategy that enables uniform Li deposition through electrostatic shielding by Na^+^ at a high Na^+^/Li^+^ molar ratio. We found that solvation energy plays a decisive role in determining deposition potentials and thus achieved the use of high relative fraction of Na^+^ shielding ions to stabilize Li deposition by electrostatic shielding in a 1-*n*-butyl-1-methylpyrrolidinium bis(trifluoromethylsulfonyl)imide ([Py_14_][TFSI])-based electrolyte. Spectral and simulation results reveal an all-anion solvation sheath where TFSI^−^ coordinates both Li^+^ and Na^+^, narrowing their solvation energy gap and thermodynamically favoring Li^+^ deposition at high Na^+^ ratio. The optimized electrolyte (0.2 M LiTFSI/0.1 M NaTFSI in [Py_14_][TFSI]) extends the cycle life of Li‖Li symmetric cells to over 1400 h at 0.2 mA cm^−2^ and enables stable Li–O_2_ battery operation for 124 cycles without capacity decay under 500 mA g^−1^. This work highlights the critical role of solvation structure in regulating deposition behavior and provides an effective approach for durable Li–O_2_ batteries.

## Abstract 2

Anion-dominated solvation supports Na^+^ shielding at Na^+^/Li^+^ = 1 : 2 and uniform Li deposition. Li‖Li cells exceed 1400 h and Li–O_2_ cells reach 124 capacity-limited cycles (500 mA g^−1^; 500 mAh g^−1^).

## Graphical abstract

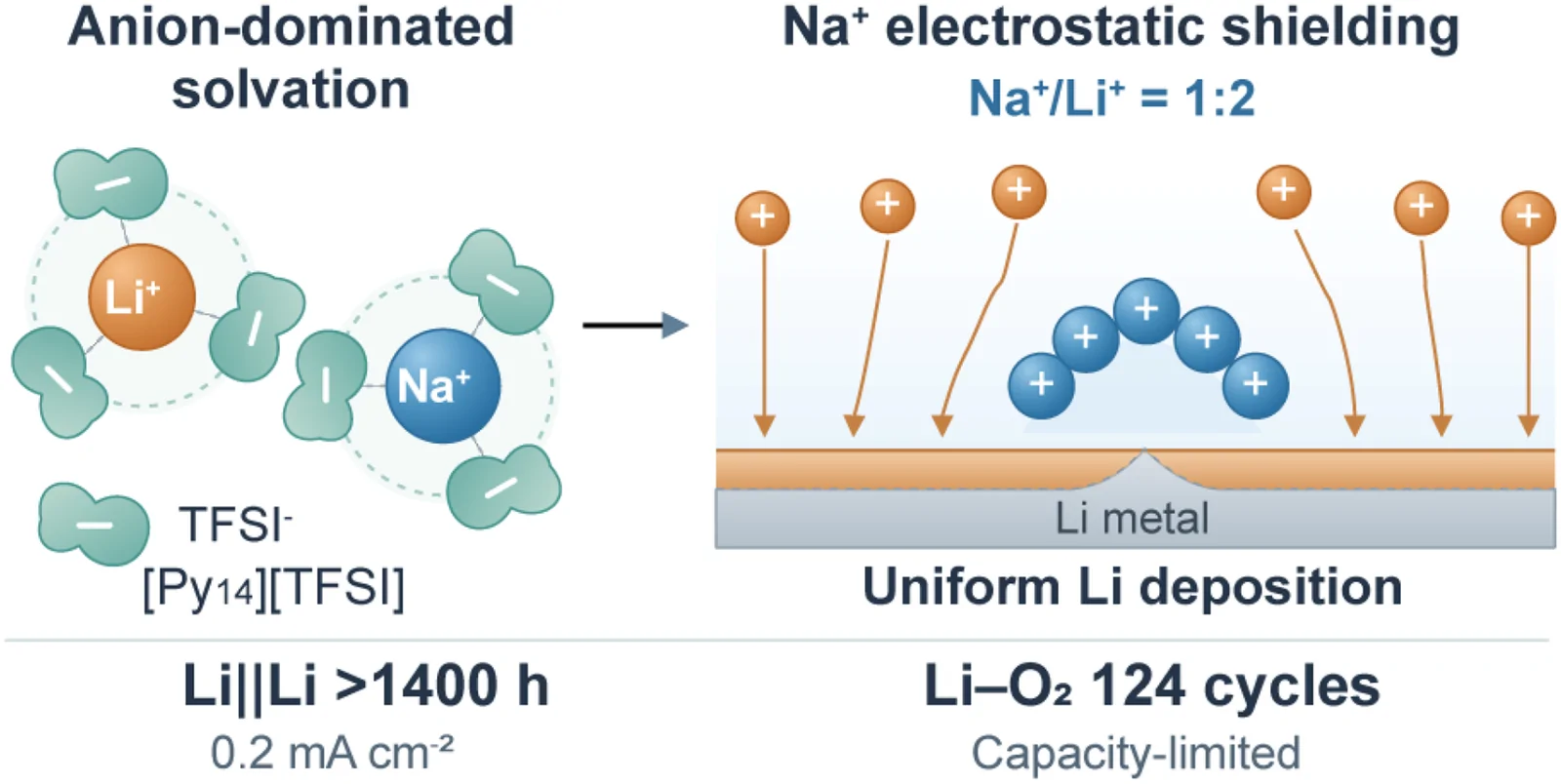

## Introduction

Aprotic lithium–oxygen (Li–O_2_) batteries have garnered significant research interest in recent years due to their exceptionally high theoretical energy density (3500 Wh kg^−1^), low cost, and environmental friendliness.^1–5^ Their electrochemical performance is fundamentally governed by the interplay between the oxygen cathode, lithium metal anode, and non-aqueous electrolyte. Despite substantial advances in cathode catalysis and electrolyte engineering to accelerate oxygen reduction and evolution reactions,^6–9^ Li-anode instability remains a critical barrier to durable Li–O_2_ batteries. Beyond repeated plating/stripping, the Li anode in oxygen-fed cells is susceptible to chemical attack by dissolved O_2_ and reactive oxygen species originating from the cathode. Nonuniform Li deposition exacerbates this vulnerability by increasing the reactive surface area, thereby promoting parasitic reactions and accelerating the consumption of active Li and electrolyte.^10–16^ This coupling between morphological instability and oxygen-induced interfacial degradation makes Li–O_2_ batteries a particularly demanding and mechanistically relevant platform for evaluating Li-deposition regulation. Achieving uniform and stable Li deposition is therefore essential to mitigating anode degradation and extending cell lifetime.

Various strategies have been developed to stabilize lithium anodes, including artificial protective films,^17^ alloy anodes,^18^ and electrolyte engineering.^19,20^ Among these, electrolyte modification represents an effective and straightforward approach for forming robust solid-electrolyte interphases (SEIs) and suppressing lithium dendrite growth.^21,22^ Among electrolyte-based approaches, the self-healing electrostatic shield (SHES) mechanism provides a distinctive means of regulating Li deposition in real time.^18,23,24^ In this mechanism, non-reducible or preferentially unreduced foreign cations accumulate near high-electric-field surface protrusions, locally repelling Li^+^ and redirecting Li deposition toward adjacent low-field regions.^18,25–33^ This dynamic response differs from static SEI regulation because newly generated protrusions can be continuously screened during repeated plating. Nevertheless, previously reported SHES strategies have mainly relied on relatively low concentrations of shielding cations, such as Cs^+^, to avoid their competitive reduction or displacement by Li metal. For example, a representative Cs^+^/Li^+^ molar ratio is approximately 1 : 20.^25,26^ Such a low shielding-ion fraction limits the interfacial population and sustained flux of shielding ions, particularly during prolonged deposition or when the electrode develops a large number of high-field nucleation sites. Increasing the relative concentration of shielding ions is therefore desirable for maintaining effective interfacial coverage, but requires strict control over their solvation and deposition thermodynamics. Moreover, the application of electrostatic shielding to realize uniform lithium deposition for high-performance Li–O_2_ batteries has not yet been systematically investigated.

Herein, we propose a solvation-energy design strategy based on the Born–Haber cycle to achieve uniform Li deposition through electrostatic shielding at a high Na^+^/Li^+^ molar ratio of 1 : 2. Na^+^ was selected as a representative shielding ion (Fig. 1a), owing to its small ionic radius and high charge density. We found that solvation energy plays a decisive role in determining deposition potentials (Fig. 1b and c). Theoretical calculations show that in a series of common solvents, the binding energy of Li^+^ is higher than that of Na^+^, whereas in 1-*n*-butyl-1-methylpyrrolidinium bis(trifluoromethylsulfonyl)imide ([Py_14_][TFSI]), the binding energy of Li^+^ with TFSI^−^ anions is lower than that of Na^+^, which is different from other solvents in Fig. 1d. Furthermore, it is found that in conventional ether solvents such as tetraethylene glycol dimethyl ether (G4), both Li^+^ and Na^+^ are primarily coordinated by solvent molecules, while TFSI^−^ anions remain free. In contrast, in [Py_14_][TFSI], both Li^+^ and Na^+^ are coordinated by TFSI^−^ anions, forming an all-anion solvation sheath. This structure significantly narrows the solvation energy difference between Na^+^ and Li^+^ (73.78 and 75.51 kJ mol^−1^, respectively), thereby altering the deposition order and enabling preferential reduction of Li^+^. Based on this reversed deposition sequence in [Py_14_][TFSI], this work successfully implements effective shielding protection for the lithium anode *via* high-concentration Na^+^ ions. By screening a wide range of Na^+^ concentrations, the optimal electrolyte composition in [Py_14_][TFSI] is identified as 0.2 M LiTFSI/0.1 M NaTFSI, which fully exploits the electrostatic shielding effect. This electrolyte significantly promotes uniform lithium deposition, extending the cycle life of Li‖Li symmetric cells from 170 h to over 1400 h at 0.2 mA cm^−2^ without noticeable polarization increase. The enhanced anode stability further ensures improved cycling durability of Li–O_2_ batteries, demonstrating promising application potential.

**Fig. 1:**
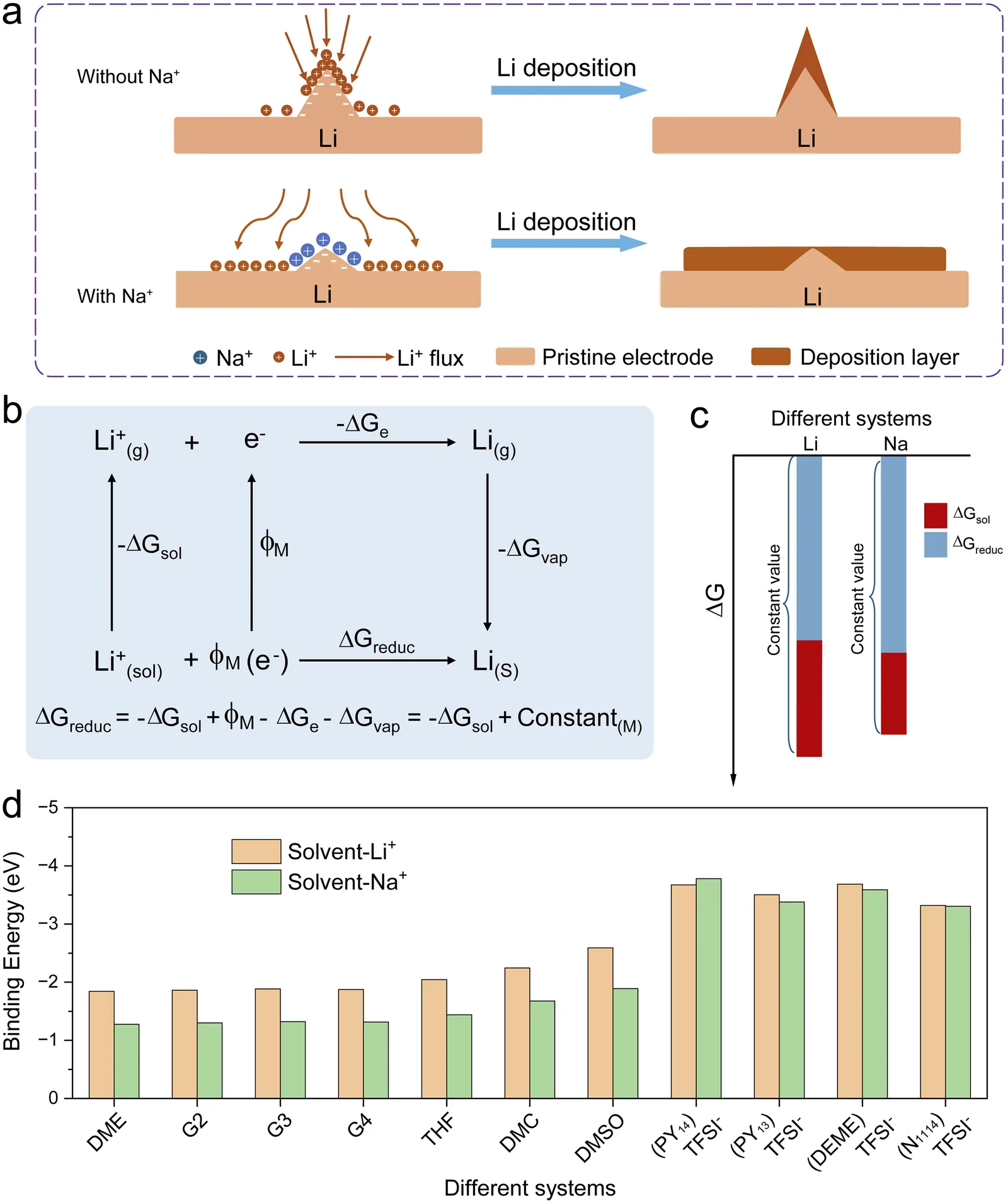
(a) Schematic illustration of the Li metal deposition behavior in electrolytes without and with Na^+^ addition. (b) Born–Haber cycle for the reduction process of a Li^+^ ion to Li metal in the electrolyte. (c) Relationship between the Li^+^ reduction energy and its solvation energy in conventional electrolyte systems. (d) Calculated binding energies of Li^+^ and Na^+^ with conventional solvent molecules and TFSI^−^ anions in representative ionic liquids.

## Results and discussion

The inherent reduction tendency of a metal is determined by the Gibbs free energy change (Δ*G*) of its reduction process. For the reduction of lithium ions from the electrolyte to metallic lithium, the Δ*G* can be calculated *via* the Born–Haber cycle (Fig. 1b), with the specific expression shown in eqn (1).^34^

1 Δ*G*_reduc_ = −Δ*G*_sol_ + *Φ*_M_ − Δ*G*_e_ − Δ*G*_vap_ = −Δ*G*_sol_ + constant_(M)_

Here, Δ*G*_reduc_ is reduction Gibbs free energy change of lithium ions from the electrolyte to metallic lithium, Δ*G*_sol_ represents the solvation energy of the lithium ion, *Φ*_M_ is the work function of lithium metal, Δ*G*_e_ is the first ionization energy of gaseous lithium atoms, and Δ*G*_vap_ is the vaporization energy of lithium. For a given metal electrode, the latter three terms are constants, thus, the key variable affecting the Δ*G*_reduc_ of the deposition process is the Δ*G*_sol_, as shown in Fig. 1c. Consequently, the relative difference in solvation energy between Li^+^ and Na^+^ in different electrolyte systems alters the Δ*G*_reduc_ of their reduction reactions, thereby dictating their reduction order.

The energy terms involved in the reduction processes of Li^+^ and Na^+^ are listed in Table S1. Disregarding solvation energy and judging solely by the first ionization energy from the perspective of the element, Li^+^ should be more readily reduced than Na^+^, consistent with the trend of increasing metallic character with atomic radius in the periodic table. However, in practical electrodeposition processes, ions are always solvated, making solvation energy the critical factor determining metal deposition behavior.

Due to its smaller ionic radius, Li^+^ typically exhibits stronger solvation than Na^+^ in conventional solvents, *i.e.*, a larger absolute solvation energy value. This renders the Δ*G* for Li^+^ reduction more positive (*i.e.*, more negative reduction potential), making it harder to reduce. To investigate the nature of solvation, we compared the binding energies of different solvents, including 1,2-dimethoxyethane (DME), diglyme (G2), triethylene glycol dimethyl ether (G3), G4, dimethyl carbonate (DMC), dimethyl sulfoxide (DMSO), and tetrahydrofuran (THF) with Li and Na, as summarized in Fig. 1d. Li^+^ shows higher binding energy with most conventional solvents than Na^+^, primarily attributable to its smaller ionic radius. From the perspective of Lewis acid–base theory, Na^+^ acts as a softer acid compared to Li^+^, and thus exhibits a stronger inherent tendency to coordinate with soft bases such as the bulky TFSI^−^ anion. Our theoretical calculations confirm that across various ionic liquid systems ([Py_14_][TFSI], *N*-methyl-*N*-propyl pyrrolidinium bis(trifluoromethanesulfonyl)imide ([Py_13_][TFSI]), *N*,*N*-diethyl-*N*-(2-methoxyethyl)-*N*-methylammonium bis (trifluoromethylsulphonyl) imide ([DEME][TFSI]), butyl-trimethylammonium bis(trifluoromethyl sulfonyl)imide ([N_1114_][TFSI])), the Na^+^–TFSI^−^ binding energy closely approaches that of Li^+^–TFSI^−^. Notably, within the [Py_14_][TFSI] system, the Na^+^–TFSI^−^ pair even demonstrates a slightly higher binding energy than the Li^+^–TFSI^−^ counterpart. This phenomenon is likely attributable to the pronounced steric hindrance exerted by the [Py_14_]^+^ cation, which reduces the interaction strength between Li^+^ and TFSI^−^ to a level lower than that of Na^+^–TFSI^−^. This distinct characteristic directly motivated our selection of [Py_14_][TFSI] as the electrolyte in this study. The resulting unique solvation structure effectively narrows the energetic gap between the solvation energies of Li^+^ and Na^+^, thereby modulating the thermodynamics of their deposition. To explore the potential extension to other alkali-metal cations, we further calculated the binding energy of K^+^ and TFSI^−^. The K^+^ binding energy with TFSI^−^ is comparable in magnitude to those of Li^+^ and Na^+^ (Fig. S1), suggesting that similar cation–anion interaction strengths are not restricted to Na^+^.

Furthermore, we experimentally determined the desolvation activation energy (*i.e.*, solvation energy) of Li/Na ions in different electrolytes, maintaining a total salt concentration of 0.3 M and an experimental temperature of 50 °C to balance conductivity and viscosity.^35^ The desolvation energies of Li^+^ and Na^+^ in the four electrolyte systems were measured using temperature-dependent electrochemical impedance spectroscopy (EIS). Li‖Li symmetric cells with 0.3 M LiTFSI/G4 (3L-G4) and 0.3 M LiTFSI/[Py_14_][TFSI] (3L-IL) electrolytes, as well as Na‖Na symmetric cells with 0.3 M NaTFSI/G4 (3N-G4) and 0.3 M NaTFSI/[Py_14_][TFSI] (3N-IL) electrolytes, were assembled and aged at 50 °C for 24 hours to allow complete passivation of the metal electrode surfaces and formation of a stable SEI. The equivalent circuit used for fitting the impedance spectra is shown in Fig. S2. The solvation energy calculation results are presented in Fig. 2a and b. In conventional solvents, the solvation energy difference between Li^+^ and Na^+^ is significant. In contrast, in the [Py_14_][TFSI] system, the solvation energy for Li^+^ is 75.51 kJ mol^−1^, while for Na^+^ it is 73.78 kJ mol^−1^, indicating they are very close. This proximity aligns with computational predictions, though a slight discrepancy is noted, the computed binding energy between TFSI^−^ and Li^+^ is marginally lower than that with Na^+^ in the [Py_14_][TFSI] system. This minor deviation likely stems from systematic uncertainties that become apparent when the two energy values are nearly equivalent. The Δ*G*_reduc_ calculated based on the Born–Haber cycle shows that in traditional G4-based electrolyte, Na^+^ reduction is thermodynamically favored over Li^+^ (Fig. S3).^29,30^ In the [Py_14_][TFSI] system, as shown in Fig. 2c, the Δ*G*_reduc_ for Li^+^ is −309.41 kJ mol^−1^, and for Na^+^ it is −289.96 kJ mol^−1^, indicating that Li^+^ deposition will occur preferentially over Na^+^.

**Fig. 2:**
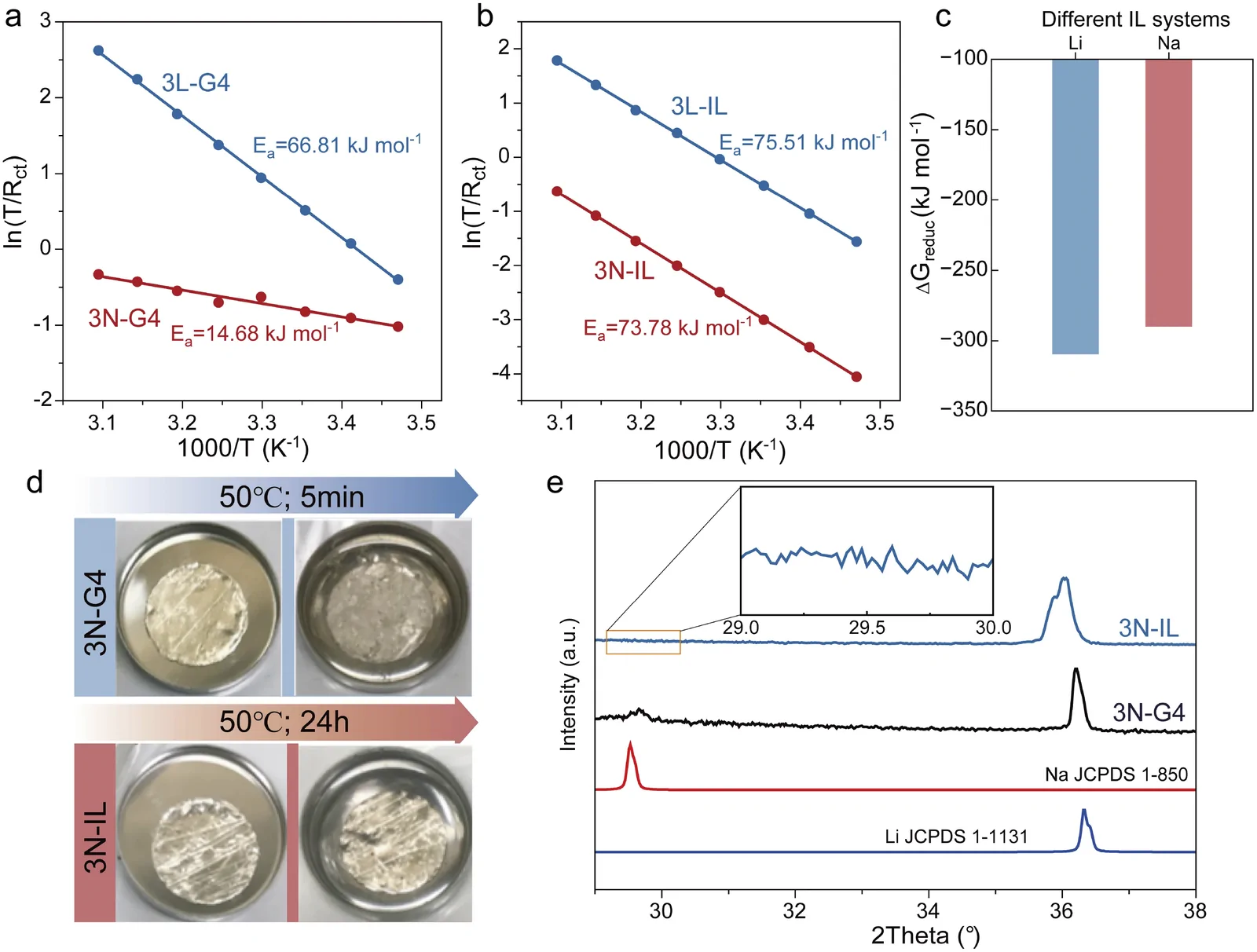
Desolvation energies of Li^+^ and Na^+^ derived from variable-temperature Nyquist plots in (a) the G4 and (b) [Py_14_][TFSI] electrolyte systems. (c) Deposition free energies of Li and Na in the [Py_14_][TFSI] system. (d) Optical images and (e) corresponding XRD patterns of a fresh lithium metal before and after immersion in different electrolytes.

To validate these calculations, we conducted displacement experiments of Na^+^ by lithium metal in different electrolytes. In 3N-G4 conventional electrolyte, the lithium foil lost its metallic luster within 5 minutes, with a dark gray substance forming on the surface, as shown in Fig. 2d. XRD patterns (Fig. 2e) reveal characteristic peaks for metallic sodium at 29.46° alongside the lithium metal peak (36.19°), confirming the existence of displacement reaction, consistent with Born–Haber cycle predictions and literature. Conversely, in 3N-IL electrolyte, even after 24 hours, the lithium foil retained a shiny surface, and no metallic sodium signal was detected by XRD, confirming the preferential reduction of Li^+^ in this system and further supporting the theoretical conclusions.

We systematically investigated the solvation environments in different electrolytes. The solvation energy of metal ions is fundamentally determined by their solvation structure in the electrolyte. Raman spectroscopy was employed to analyze changes in solvation structure. As shown in Fig. 3a, the S–N–S vibration of TFSI^−^ in pure [Py_14_][TFSI] (IL) appears as a single peak at 740.4 cm^−1^, indicating weak association between TFSI^−^ and [Py_14_]^+^ cations. In the 3L-IL electrolyte, besides the peak corresponding to free TFSI^−^, a new peak emerged at 746.7 cm^−1^, attributable to the characteristic signal of TFSI^−^ forming contact ion pairs (CIPs) with Li^+^.^36^ Since TFSI^−^ anions are the only species available to coordinate Li^+^ in this system, it can be inferred that Li^+^ is solely coordinated by TFSI^−^ in a CIP mode. At low lithium salt concentrations in TFSI-ILs, each Li^+^ tends to form a [Li(TFSI)_2_]^−^ complex with two TFSI^−^ anions, fitting the CIP coordination model.^37^ Therefore, aside from the TFSI^−^ coordinating Li^+^, a large number of free TFSI^−^ anions remain in the system, as evidenced by the large peak area at this position (740.4 cm^−1^) in the Raman deconvolution results.

**Fig. 3:**
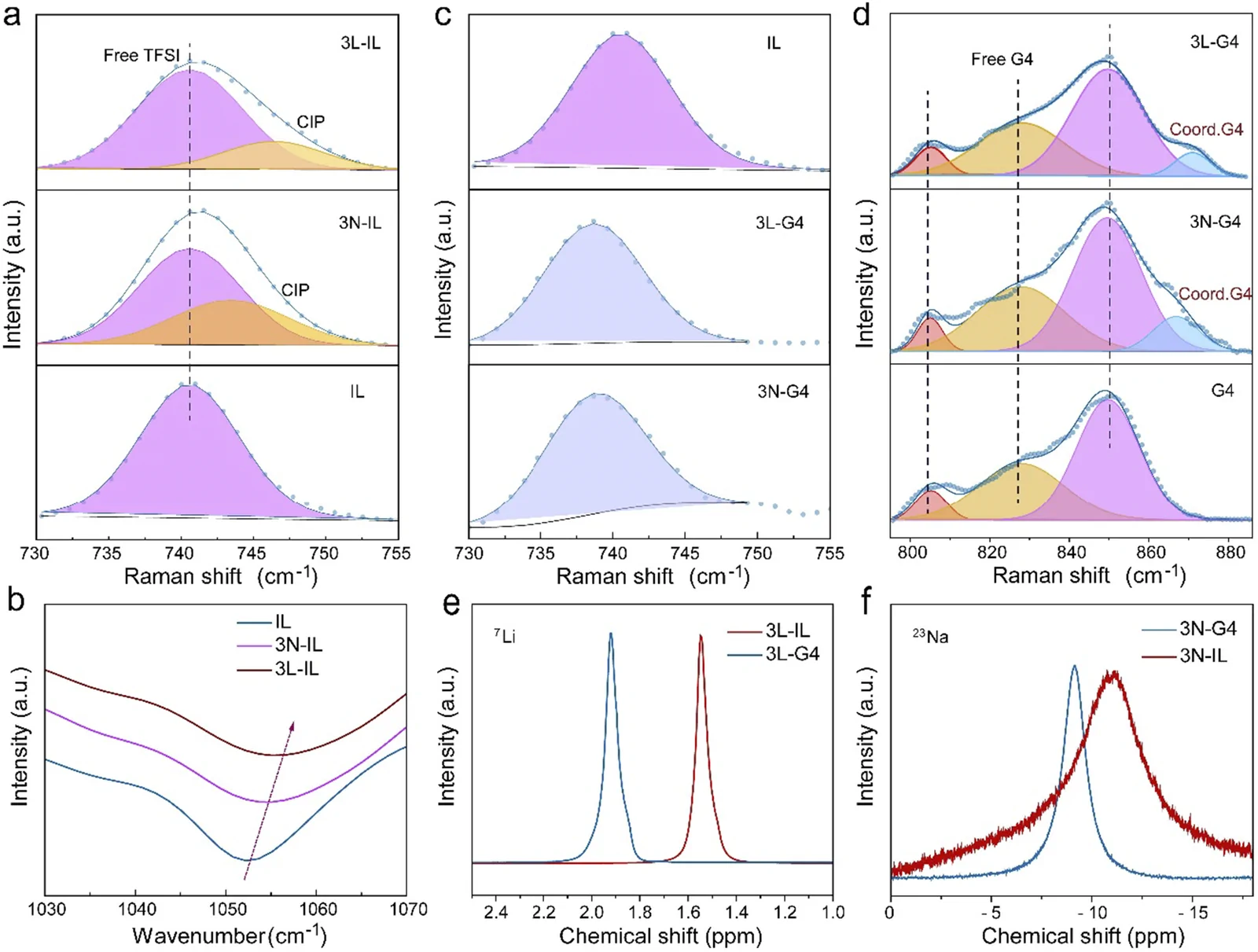
Spectroscopic characterization of the different electrolytes. (a) Raman spectra and deconvolution of the S–N–S vibration in 3L-IL, 3N-IL, and pure [Py_14_][TFSI]. (b) FTIR spectra of the S–N–S vibration in 3L-IL, 3N-IL, and pure [Py_14_][TFSI]. (c) Comparison of the S–N–S Raman signals in 3L-G4, 3N-G4, and pure [Py_14_][TFSI]. (d) Raman spectra and deconvolution of the C–O–C vibration in 3L-G4, 3N-G4, and pure G4. (e) ^7^Li NMR spectra of 3L-G4 and 3L-IL. (f) ^23^Na NMR spectra of 3N-G4 and 3N-IL.

A similar phenomenon was observed in the 3N-IL electrolyte. The experimentally determined solvation energies reveal that Li^+^ exhibits slightly higher solvation energy than Na^+^ in the [Py_14_][TFSI] system. Consistent with this trend, the lower charge density of Na^+^ leads to weaker binding with TFSI^−^ compared to Li^+^, as reflected in the corresponding CIP peak appearing at a slightly lower wavenumber (745.0 cm^−1^). This spectral feature confirms that Na^+^ is also coordinated by TFSI^−^, while indicating the presence of abundant uncoordinated TFSI^−^ anions in the electrolyte. This conclusion is further supported by FTIR spectra (Fig. 3b). The S–N–S vibration peak of TFSI^−^ in pure [Py_14_][TFSI] is at 1052.4 cm^−1^, shifting to 1054 cm^−1^ upon adding 0.3 M NaTFSI, indicating coordination between Na^+^ and TFSI^−^. In the 3L-IL system, this peak further blue-shifts to 1055.1 cm^−1^, reflecting stronger coordination between Li^+^ and TFSI^−^.

The solvation structure in G4-based electrolytes was further compared. In both 3L-G4 and 3N-G4, the S–N–S Raman peak positions of TFSI^−^ are lower than that of free TFSI^−^ in pure [Py_14_][TFSI] and completely overlap (Fig. 3c), indicating that TFSI^−^ remains entirely free in the G4 system, not participating in cation coordination, and its vibrational behavior is independent of cation type.^38^ Additionally, Raman spectra of the G4 molecule's C–O–C vibration show, besides the three characteristic peaks of free G4 (805.2, 828.2, and 849.6 cm^−1^), a new high-wavenumber peak appears in both 3L-G4 and 3N-G4, corresponding to G4 molecules coordinated to cations, as shown in Fig. 3d. Due to the higher charge density of Li^+^, the C–O–C peak of coordinated G4 exhibits a more pronounced blue shift (870.8 cm^−1^).^39^

^7^Li NMR and ^23^Na NMR were used to further directly verify the coordination environment. As shown in Fig. 3e, the ^7^Li signal in 3L-IL shifts upfield compared to the G4 system, with peak broadening due to increased viscosity. Similarly, as shown in Fig. 3f, the ^23^Na signal in 3N-IL also shifts upfield (−11.06 ppm *vs.* −9.14 ppm in the G4 system), demonstrating that the shielding effect of TFSI^−^ anions on metal ions in [Py_14_][TFSI] is stronger than that of G4 molecules, resulting in lower chemical shifts.

Based on the above spectroscopic analyses, in G4-based electrolytes, Li^+^ and Na^+^ are predominantly coordinated by G4 molecules, with TFSI^−^ in a free state. In contrast, in [Py_14_][TFSI]-based electrolytes, each metal ion is coordinated by at least two TFSI^−^ anions, while the remaining [Py_14_][TFSI] stays free. The difference in solvation structure directly influences the solvation energy of Li^+^ and Na^+^, thereby regulating the free energy change of their deposition reactions. Molecular dynamics simulations (Fig. 4 and S4) further corroborate that in [Py_14_][TFSI] systems containing Li^+^ and Na^+^, the coordination between the cations and TFSI^−^ remains dominant. While Li^+^ in the G4 system is primarily solvated by solvent molecules, it mainly coordinates with TFSI^−^ anions in the [Py_14_][TFSI] system. Radial distribution function (RDF) analyses indicate that Li^+^ is coordinated by three TFSI^−^ anions in the [Py_14_][TFSI] system, as shown in Fig. 4a and c, in contrast to the reported coordination with only two TFSI^−^ anions in the [DEME][TFSI] system.^37^ This difference suggests that the [Py_14_]^+^ cation weakens the coordinating ability of individual TFSI^−^ anions, requiring more anions to effectively solvate Li^+^. This further demonstrates the advanced capability of [Py_14_][TFSI] in achieving full anion-based coordination.

**Fig. 4:**
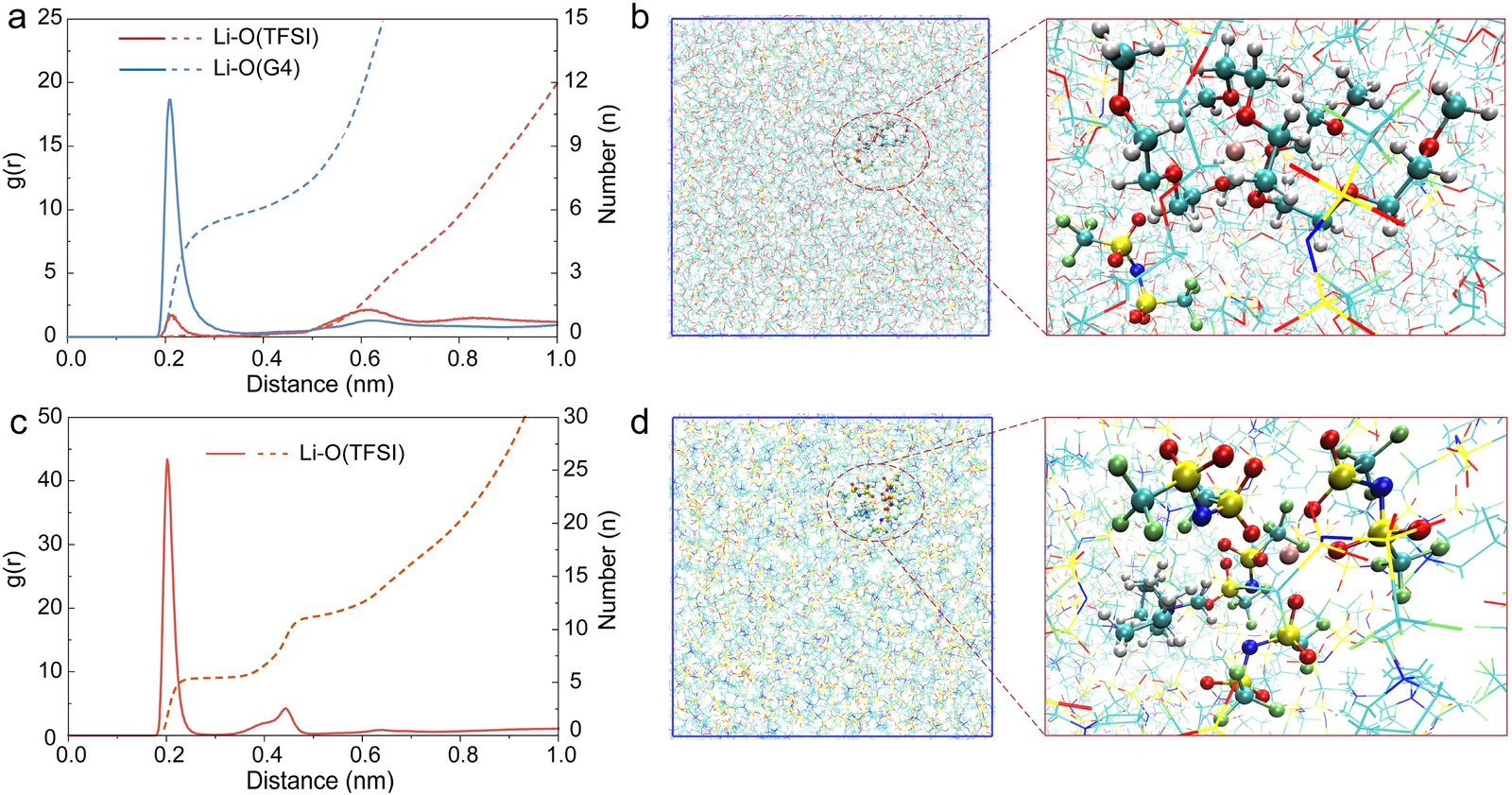
RDFs (solid lines) and coordination number (dashed lines) of (a) Li–O (TFSI, G4) for 3L-G4 and (c) Li–O (TFSI) for 3L-IL electrolyte. MD simulation snapshots of Li^+^ solvation sheath in (b) 3L-G4 and (d) 3L-IL electrolyte. (white ball: H; red ball: O; green ball: F; cyan ball: C; blue ball: S; yellow ball: N; purple ball: Li).

Thus, the spectroscopic and simulation results consistently demonstrate strong cation-TFSI^−^ association and an anion-dominated first solvation shell in the [Py_14_][TFSI]-based electrolytes. This coordination environment narrows the difference between the solvation characteristics of Li^+^ and Na^+^, thereby enabling Li^+^ reduction to remain thermodynamically preferred over Na^+^. The resulting deposition selectivity provides the prerequisite for employing Na^+^ as an electrostatic shielding ion over a broad Na^+^/Li^+^ composition range.

To determine the optimal Na^+^ addition amount for protecting the lithium metal electrode, electrolyte systems with different Na^+^/Li^+^ ratios were selected for study, specifically including: 0.3 mol L^−1^ LiTFSI in [Py_14_][TFSI] (3L-IL), 0.25 mol L^−1^ LiTFSI + 0.05 mol L^−1^ NaTFSI in [Py_14_][TFSI] (5L1N-IL), 0.225 mol L^−1^ LiTFSI + 0.075 mol L^−1^ NaTFSI in [Py_14_][TFSI] (3L1N-IL), 0.2 mol L^−1^ LiTFSI + 0.1 mol L^−1^ NaTFSI in [Py_14_][TFSI] (2L1N-IL), and 0.15 mol L^−1^ LiTFSI + 0.15 mol L^−1^ NaTFSI in [Py_14_][TFSI] (1L1N-IL). These electrolytes correspond to Na : Li molar ratios ranging from 0 : 1 to 1 : 1.

First, the deposition products in the four sodium-containing electrolytes were characterized by XRD. As shown in Fig. 5a, all deposition products were metallic lithium, with no signals of metallic sodium detected, further confirming that in the [Py_14_][TFSI] electrolyte system with mixed Li/Na salts, lithium metal deposits preferentially over sodium metal, satisfying the prerequisite for Na^+^ electrostatic shielding.

**Fig. 5:**
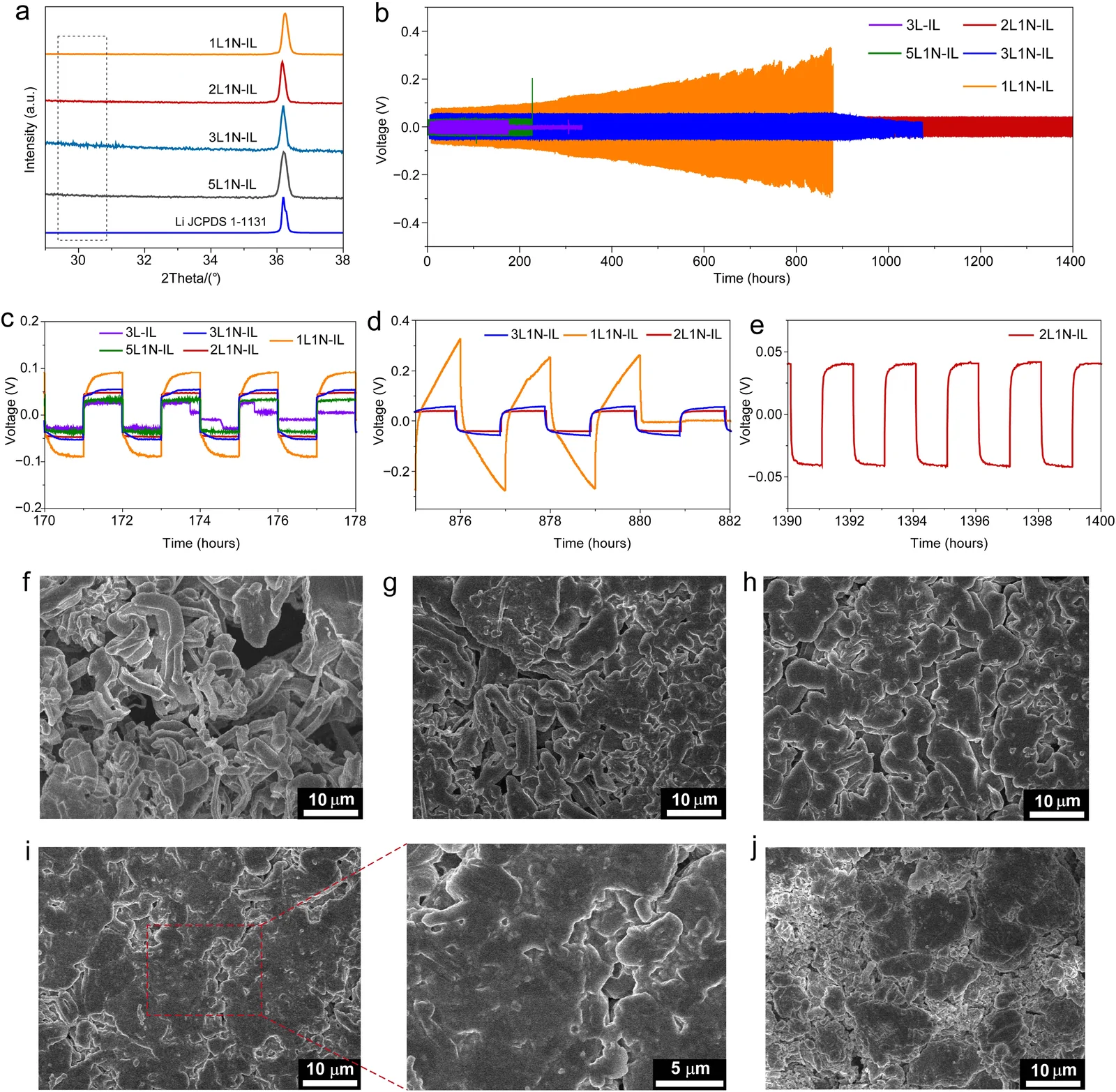
(a) XRD pattern of the deposition products on lithium electrode from the sodium-containing electrolyte. (b) Voltage profiles of Li‖Li symmetric cells employing different electrolytes during cycling at 0.2 mA cm^−2^. (c–e) Voltage profiles at different time intervals. Surface morphology of a lithium electrode after depositing 5 mAh cm^−2^ at 0.2 mA cm^−2^ in the different electrolyte: (f) 3L-IL, (g) 5L1N-IL, (h) 3L1N-IL, (i) 2L1N-IL, (j) 1L1N-IL.

To further optimize the Na^+^ additive ratio, the electrochemical performance of Li‖Li symmetric cells was evaluated. As seen in Fig. 5b–e, although the cell employing the 2L1N-IL electrolyte exhibited a slightly higher initial overpotential compared to that using 3L-IL (42 mV *vs.* 27 mV), the 3L-IL cell experienced a short circuit within 180 h. In contrast, the 2L1N-IL cell, which contains a higher Na^+^ concentration, demonstrated significantly enhanced stability and cycled continuously for over 1400 h without failure. Enlarged segments of the voltage time curves show that both cells initially operated stably. The 3L-IL cell, however, exhibited a sudden voltage drop at approximately 170 h, followed by a brief recovery at approximately 171 h. This behavior is consistent with a transient micro-short circuit potentially associated with dendritic Li growth, although it does not by itself provide definitive evidence of separator penetration or dendrite fusion. The subsequent persistent voltage collapse at approximately 172 h indicates eventual cell failure. In contrast, symmetric cells employing shielding-ion electrolytes demonstrated markedly extended cycle life. Notably, the 2L1N-IL cell maintained highly stable voltage profiles throughout cycling, with no significant overpotential increase even after 1400 h. This performance surpasses the best-reported results of ionic-liquid-based lithium anode protection in Li–O_2_ batteries (Table S2). This enhanced stability is consistent with the more uniform Li deposition enabled by the electrostatic shielding effect.

To examine the electrochemical response beyond this baseline condition, Li‖Li symmetric cells were additionally tested at sequentially increasing current densities of 0.1, 0.2, and 0.5 mA cm^−2^ (Fig. S5). At 0.5 mA cm^−2^, the cell with 2L1N-IL exhibits lower voltage polarization and less pronounced polarization growth than the cell with 3L-IL over the measured interval. These results show that the beneficial effect of the optimized electrolyte extends to the higher current density examined. Together with the cycling duration exceeding 1400 h at 0.2 mA cm^−2^, the additional measurements support improved Li plating/stripping stability over the investigated operating conditions.

To correlate Na^+^ content with protective performance, the morphology of lithium deposited at 0.2 mA cm^−2^ to a capacity of 5 mAh cm^−2^ was examined by SEM (Fig. 5f–j and S6). SEM images revealed that in the 3L-IL electrolyte, the lithium deposition morphology consisted of uneven, dendritic, twisted aggregates, forming a porous deposition layer (Fig. 5f). Upon introducing Na^+^, the uneven deposition was alleviated, the proportion of curved lithium dendrites significantly decreased, and the voids in the deposition layer were substantially compressed (Fig. 5g–j). Even a small amount of added Na^+^ effectively suppressed lithium dendrite growth *via* its electrostatic shielding effect. As the Na^+^ ratio increased further, dendrites nearly disappeared, replaced by chunk-like lithium with sizes approximately 5 µm and smaller inter-particle voids. In the 2L1N-IL electrolyte, the lithium deposition layer exhibited a dense and flat morphology (Fig. 5i). Further increasing the Na^+^ fraction to 1 : 1, however, decreases the bulk Li^+^ concentration and promotes local Li^+^ depletion,^37,40^ resulting in the reappearance of nonuniform deposits (Fig. 5j). These results demonstrate that effective electrostatic shielding requires a balance between the interfacial availability of shielding ions and sufficient Li^+^ transport. Thus, the significance of the present solvation-mediated strategy lies in expanding the accessible shielding-ion concentration range while retaining selective Li deposition, rather than simply maximizing the Na^+^.

Temperature-dependent conductivity measurements reveal that 2L1N-IL exhibits slightly lower ionic conductivity than 3L-IL near room temperature, but higher conductivity at elevated temperatures (Fig. S7). The corresponding apparent activation energies are 34.76 and 27.62 kJ mol^−1^, respectively, indicating a stronger temperature dependence of ionic conduction in 2L1N-IL. Notably, the improved electrochemical performance of 2L1N-IL, despite its lower near-ambient conductivity, suggests that Na^+^-mediated interfacial regulation, rather than enhanced bulk ionic conduction, plays an important role in stabilizing Li deposition.

Finite element simulation was further employed to compare the effects on lithium deposition behavior with and without electrostatic shielding. The exchange current densities for lithium deposition/stripping in the two electrolytes were first measured. The exchange current density in the 2L1N-IL electrolyte containing shielding ions was significantly lower than that in the 3L-IL electrolyte without shielding ions (0.195 *vs.* 0.651 mA cm^−2^, Fig. S8). This phenomenon may be attributed to the preferential adsorption of Na^+^ at high-surface-area electrode tips, inhibiting lithium deposition at these sites and manifesting as slower deposition/stripping kinetics. Based on these experimentally consistent results and adhering to literature settings for electrostatic shielding, lithium deposition behavior was simulated.^41^ We constructed a model cell possessing initial electrode surface with a protruding tip to simulate the unavoidable surface convex parts of pristine Li electrode in reality or the Li nucleation sites formed during deposition (Fig. S9). The deposition simulation in different electrolytes was conducted for 1 hour at current density of 1 mA cm^−2^. As shown in Fig. 6a, results show that in the electrolyte without shielding ions, the deposition current was primarily concentrated at the electrode tip protrusion, with very low current density at the root of the protrusion and the electrode plane. This nonuniform current distribution promotes lithium metal deposition at the tips of original protrusion, promoting its further upward growth and forming an uneven deposition morphology. In contrast, in the electrolyte containing electrostatic shielding ions, the depositing ion flux was uniformly distributed across the entire electrode surface rather than concentrating at the protrusion. Consequently, lithium deposition could occur at both the protrusion root and the planar regions, thereby repairing and smoothing the initial electrode's protrusive morphology.

**Fig. 6:**
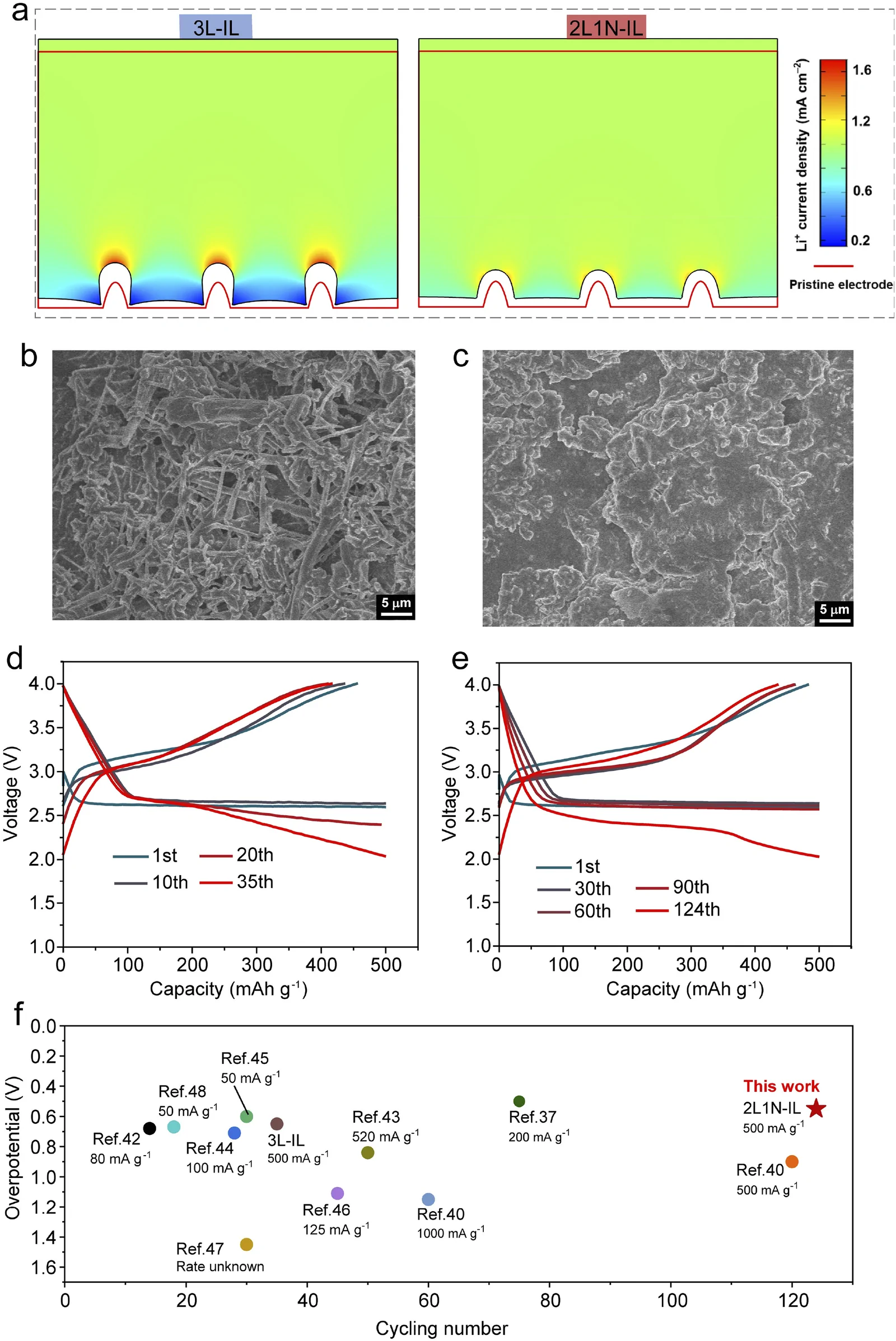
(a) Comparison of simulated lithium deposition behaviors in electrolytes without and with electrostatic shielding ions. (b and c) Surface morphologies of the lithium electrodes from Li‖Li symmetric cells after 20 cycles using (b) the 3L-IL and (c) the 2L1N-IL electrolyte. (d and e) Selected discharge–charge profiles of Li–O_2_ cells using (d) 3L-IL and (e) 2L1N-IL at 500 mA g^−1^ under a capacity-limited protocol with a cutoff capacity of 500 mAh g^−1^. (f) Electrochemical performance comparison between the Li–O_2_ battery in this work and reported systems employing TFSI-IL electrolytes.

Morphological analysis of the cycled lithium electrodes revealed that after 10 cycles in the 3L-IL electrolyte, the electrode surface shows residual short, fine dendrites and signs of pulverization (Fig. S10a), consistent with the dendritic morphology formed during the first deposition in this electrolyte. Such fine dendrites are prone to form “dead lithium” detached from the main electrode body during stripping, leaving behind substantial SEI residue. As cycling continued, the length of the dendritic residues on the electrode surface further increased (Fig. 6b), indicating aggravated “dead lithium” accumulation and decreased lithium deposition utilization. The continuous accumulation of these elongated dendritic residues may increase the probability of transient micro-short circuits and contribute to the eventual cell failure.

In contrast, the lithium electrode cycled in the 2L1N-IL electrolyte maintained an overall flat and dense surface throughout the cycling process, with no observed dendrites or their residues (Fig. 6c and S10b). This morphological evolution can be attributed primarily to the more uniform and compact Li deposition observed in the 2L1N-IL electrolyte, making it less likely to generate “dead lithium” and SEI residue during stripping, thereby preserving the post-cycling electrode flatness and ultimately contributing to the long-term stable cycling of the symmetric cell.

To examine the retention of Na-containing species at the cycled Li interface, *ex situ* XPS was performed on a Li electrode recovered from a Li‖Li symmetric cell after 20 cycles in 2L1N-IL (Fig. S11). The spectra reveal LiF and ionic-liquid/TFSI-related species, whereas no discernible Na 1s peak is detected under the employed acquisition conditions. This observation is compatible with the proposed electrostatic screening mechanism, which does not require permanent incorporation of Na into the SEI.

Li–O_2_ cells were assembled to evaluate the contribution of Li-anode stabilization to full-cell performance. All cells were cycled at 500 mA g^−1^ under a capacity-limited protocol with a cutoff capacity of 500 mAh g^−1^. Both [Py_14_][TFSI]-based cells initially exhibited relatively low polarization, which may partly reflect the stabilizing effect of the ionic liquid on superoxide intermediates (O_2_^−^). However, the cell using 3L-IL showed rapid voltage and capacity decay within 35 cycles (Fig. 6d). In contrast, the 2L1N-IL cell sustained 124 cycles while maintaining relatively low polarization (Fig. 6e). These results support the role of Li-anode stabilization in mitigating polarization growth and extending the capacity-limited cycling life of Li–O_2_ batteries. The electrochemical performance of the Li–O_2_ battery was systematically compared with previously reported systems based on TFSI-IL electrolytes.^37,40,42–48^ As shown in Fig. 6f and Table S2, the battery employing the optimized electrolyte 2L1N-IL demonstrates the improved cycle lifetime among reported counterparts, while also maintaining low cycling overpotential. More importantly, most previous ionic-liquid-based strategies primarily improve Li–O_2_ performance through SEI formation, redox-mediator regulation, or modification of cathodic oxygen chemistry. In contrast, the present work regulates the relative deposition behavior of Li^+^ and Na^+^ through their solvation environments, allowing a high relative fraction of Na^+^ to provide dynamic electrostatic shielding without detectable Na-metal formation. Therefore, the conceptual advance lies in establishing a direct connection among solvation regulation, cation deposition selectivity, electrostatic shielding, and Li-anode stabilization under an oxygen-containing environment.

To investigate the cause of failure in battery based on the 3L-IL electrolyte, the failed battery was disassembled, its lithium anode replaced with a fresh lithium foil, reassembled, and continued cycling under the same conditions. The results show that the original battery suffered a sharp discharge capacity drop before 20 cycles, whereas after anode replacement, the battery immediately recovered to the cut-off discharge capacity and continued stable cycling for over ten more cycles (which is almost as long as the fresh Li–O_2_ battery with 3L-IL electrolyte) (Fig. S12). This indicates that the cell failure primarily originated from lithium anode failure, not cathode performance degradation.

Morphological characterization of the lithium anode retrieved from the failed cell revealed its surface was covered with extensive dendritic residues (Fig. S13). At the Li–O_2_ battery operating temperature, the reduced viscosity of the IL electrolyte facilitates easier diffusion of dissolved oxygen to the anode, where it reacts with the high-surface-area fine dendritic lithium, accelerating electrode failure. This phenomenon aligns with literature reports of exacerbated lithium anode pulverization in oxygen environments.^49^ SEM-EDS mapping analysis (Fig. S14) further shows that the dendritic residues mainly consisted of C, N, O, F, and S elements, corresponding to decomposition products of the TFSI^−^ anion. The high oxygen content (Fig. S15 and Table S3) corroborates the speculation that oxygen participates in and exacerbates side reactions. Therefore, achieving uniform and stable deposition of the lithium anode is crucial for enhancing the overall cycling stability of Li–O_2_ batteries.

## Conclusions

In summary, this study establishes a solvation-energy design strategy that enables uniform Li deposition through Na^+^-mediated electrostatic shielding at a high Na^+^/Li^+^ molar ratio of 1 : 2. In-depth analysis of the solvation structure in the [Py_14_][TFSI] electrolyte revealed that its unique all-anion solvation environment significantly narrows the solvation energy difference between Li^+^ and Na^+^. This results in a more negative free energy change for the reduction of Li^+^ to metallic lithium compared to Na^+^, thereby enabling preferential lithium deposition over sodium. The optimized 2L1N-IL electrolyte significantly enhances the cycling stability of Li‖Li symmetric cells, extending their lifespan to over 1400 hours. In the Li–O_2_ battery system, the 2L1N-IL electrolyte enables superior cycling performance compared to those using the Na^+^-free 3L-IL electrolyte, achieving 124 cycles without capacity decay while maintaining low overpotential, primarily attributable to the significantly improved anode interface stability. This work provides new insights and experimental evidence for stabilizing Li–O_2_ batteries through electrolyte design.

## Author contributions

Z. Y. and Y. C. designed the project. Z. Y. and Y. C. performed all the experiments including characterization and electrochemical tests. S. X. conducted theoretical calculations. Z. Y., Y. C. and Y. L. carried out the analyses. J. C. supervised the project. All authors discussed the results and contributed to the preparation of the manuscript.

## Conflicts of interest

There are no conflicts to declare.

## Supplementary Material

SC-OLF-D6SC06234B-s001

## Data Availability

The data supporting this article have been included in the article and its supplementary information (SI). Additional data are available from the corresponding author upon reasonable request. Supplementary information is available. See DOI: https://doi.org/10.1039/d6sc06234b.
