# Supplementary material for: Solvation-mediated electrostatic shielding of Li anodes by Na+ for durable lithium–oxygen batteries
Source: Chem Sci. 2026 Oct 27. Online ahead of print. doi: 10.1039/d6sc06234b (PMC13728931; doi:10.1039/d6sc06234b)
Supplement: Supplementary file 1 [file SC-OLF-D6SC06234B-s001.pdf]

Supplementary Information (SI) for Chemical Science.
This journal is © The Royal Society of Chemistry

Supporting Information

Solvation-Mediated Electrostatic Shielding of Li Anodes by Na^+ for Durable Lithium–Oxygen Batteries

Zhuo Yang†, Yichao Cai†, Shuo Xu, Yong Lu, Jun Chen*

Key Laboratory of Advanced Energy Materials Chemistry (Ministry of Education), State Key Laboratory of Advanced Chemical Power Sources, College of Chemistry, Nankai University, Tianjin 300071, China

*Correspondence: Jun Chen: chenabc@nankai.edu.cn

† Z. Y. and Y. C. contributed equally to this work.

Materials

Tetraethylene glycol dimethyl ether (G4), lithium bis(trifluoromethanesulfonyl)imide (LiTFSI) and sodium bis(trifluoromethanesulfonyl)imide (NaTFSI) were purchased from DoDoChem. 1-Methyl-1-butylpyrrolidinium bis(trifluoromethanesulfonyl)imide ([Py_{14}][TFSI]) was purchased from Lanzhou Institute of Chemical Physics.

Material characterization

X-ray diffraction (XRD) was employed to characterize the metallic lithium electrodes after immersion or deposition, with the aim of detecting potential metallic sodium in the surface phases. The morphological features of the lithium metal surfaces following deposition or cycling were characterized by cold-field emission scanning electron microscopy (SEM). Fourier-transform infrared (FTIR) spectroscopy and Raman spectroscopy were used to probe the coordination of Li^+ and Na^+ with $TFSI^-$ anions or G4 molecules. Additionally, ^7Li and ^{23}Na nuclear magnetic resonance (NMR) spectroscopy were utilized to characterize the coordination environments of Li^+ and Na^+. X-ray photoelectron spectroscopy (XPS) was performed using an ESCALAB 250Xi spectrometer (Thermo Fisher Scientific) on a cycled Li electrode.

Electrochemical measurements

Cells were assembled in an Ar-filled glovebox (H_2O and O_2 < 0.1 ppm). LiTFSI and NaTFSI were dried at 110 °C under Ar, and [Py_{14}][TFSI] was stirred under vacuum at 95 °C

for >48 h.

Li||Li cells were assembled in sealed CR2032 cases using Li disks (12 mm diameter), Celgard 2500 separators (16 mm diameter), and 50 µL of electrolyte. Li–O_2 cells used CR2032 cases with cathode-side openings, a Li anode, a glass-fiber separator, and a Ketjenblack-coated carbon-paper cathode.

To prepare the cathode, sodium carboxymethyl cellulose (CMC) was ultrasonically dispersed in ultrapure water, followed by addition of ethanol and Ketjenblack (Ketjenblack/CMC mass ratio, 9:1) and sonication for 4 h. The slurry was sprayed onto carbon paper at 100 °C under Ar and vacuum-dried at 100 °C for 10 h.

Li–O_2 cells were placed in 250 mL glass bottles and exposed to O_2 after three evacuation/refilling cycles using a dual-manifold system. Galvanostatic cycling was performed using a LAND battery tester; all electrochemical tests were conducted at 50 °C unless otherwise specified. Li–O_2 cycling used a current density of 500 mA g^{-1}, a discharge capacity limit of 500 mAh g^{-1}, and voltage cutoffs of 2.0 and 4.0 V.

Long-term Li||Li cycling was evaluated in both electrolytes at 0.2 mA cm^{-2} and 0.2 mAh cm^{-2} per half-cycle. Additional tests sequentially applied current densities of 0.1, 0.2, and 0.5 mA cm^{-2}.

Ionic conductivities of both electrolytes were measured from 25 to 50 °C in 5 °C increments after equilibration for 60 min at each temperature:

$$\sigma = \frac{L}{RA} \qquad \text{(S1)}$$

where L, A, and R are the electrode separation, effective electrode area, and bulk electrolyte resistance, respectively. The apparent activation energy for ionic conduction (E_a) was obtained by Arrhenius fitting:

$$\ln \sigma = \ln \sigma_0 - \frac{E_\mathrm{a}}{R} \cdot \frac{1}{T} \qquad \text{(S2)}$$

where σ_0 is the pre-exponential factor, T is the absolute temperature, and R is the gas constant (8.314 J mol^{-1} K^{-1}).

Temperature-dependent electrochemical impedance spectroscopy (EIS) was performed on Li||Li cells containing 0.3 M LiTFSI in G4 or [Py_{14}][TFSI] and Na||Na cells containing 0.3 M NaTFSI in the same solvents. Cells were aged at 50 °C for 24 h before measurements at 15–

50 °C in 5 °C increments. Spectra were fitted using the equivalent circuit in Figure S2, with R_0, R_1, and R_2 assigned to ohmic resistance, ion transport through the solid electrolyte interphase (SEI), and interfacial charge transfer involving desolvation, respectively. The apparent interfacial activation energy (E_a) was extracted using:

$$\ln\left(\frac{T}{R_2}\right) = C - \frac{E_a}{RT} \qquad (S3)$$

where C is a constant. Linear fitting of $\ln(T/R_2)$ versus 1000/T gives $E_a = -1000Rm$, where m is the slope and E_a is expressed in J mol^{-1}.

Modeling and Simulation

Finite element simulations were performed to investigate the lithium deposition behavior in electrolytes with and without Na^+ additives. The analysis focused primarily on the morphological evolution of the electrode surface and the current density distribution at the electrolyte-electrode interface.

The battery model comprised a counter electrode, an electrolyte domain, and a working electrode featuring an initial protrusion. The model had a total width of 14 μm. The distance from the counter electrode to the base plane of the working electrode was 10 μm, with the protrusion height being 1 μm. The space between the two electrode surfaces was defined as being filled with the electrolyte.

The simulations employed a tertiary current distribution model, coupled with a deformed geometry interface. The ionic transport in the electrolyte during deposition was described by the Nernst-Planck equation for dilute species. To account for the presence of a separator in a real cell, the convective velocity within the equation was set to zero, simplifying it to:

$$N_i = -\mathrm{D}_i \nabla \mathrm{c}_i - z_i u_{m,j} \mathrm{F} \mathrm{c}_i \nabla \Phi_i$$

In this equation, the first term represents ion diffusion driven by the concentration gradient, while the second term represents ion migration due to the electric field force. The deformation of the metal electrode surface was modeled based on Faraday's law. The corresponding volumetric change of lithium metal for different deposition charges was calculated using its atomic weight and density.

Computational Methods

The geometry optimizations, frequency calculations levels were carried out using Gaussian 16.A.03 package[1] with B3LYP-D3(BJ) functionals. Molecular dynamics simulations were performed using GROMACS 2023 package.[2, 3] The electrolyte systems contain 454 G4 molecules, 30 LiTFSI (NaTFSI) molecules to mimic 0.3M LiTFSI (NaTFSI)/G4 electrolytes. At the same time, we have constructed [Py_{14}][TFSI] system as a comparison, which contains 100 [Py_{14}][TFSI] molecules and 30 LiTFSI (NaTFSI) molecules. The force field parameters for solvents and ions were derived from the general AMBER force field (GAFF),[4, 5] and the MD parameters were generated by ACPYPE script,[6] where the atomic charges were generated from restrained fitting on the electrostatic potential (RESP).[7-9] The atomic charges of ions were scaled down by a factor of 0.7 to account for polarization. After being minimized by the steepest-descent algorithm, the systems were further equilibrated in the NPT ensemble using a thermal annealing approach with a total time of 5 ns. The temperature of systems was initially raised from 0 K to 500 K in a period of 1 ns. After being maintaining at 500 K for 1 ns, the systems were cooled down to 300 K within 1 ns and equilibrated at 300 K for an additional 2 ns. Subsequently, a 15 ns NPT simulation was conducted to sample the structures for data analysis. The temperature was controlled by the V-rescale thermostat with a damping time constant of 0.3 ps, while the pressure was maintained at 1 bar using the C-rescale barostat with a time constant of 2 ps. The cutoffs for the short-range electrostatic and van der Waals interactions were set to 1.2 nm. Radial distribution functions (RDF) were calculated by GROMACS package. Snapshots of MD simulation was produced with VMD software.[10]

Table S1. Gibbs Free Energy Change and Associated Energy for Li^+ and Na^+ in Electron-Accepting Deposition Processes

	ΔG_{sol} (kJ mol^{-1})	Φ_M (kJ mol^{-1})	ΔG_e (kJ mol^{-1})	ΔG_{vap} (kJ mol^{-1})	ΔG_{reduc} (kJ mol^{-1})
Li	–x	278.4	517.4	145.92	–384.92+x
Na	–y	226.56	493.34	96.96	–363.74+y

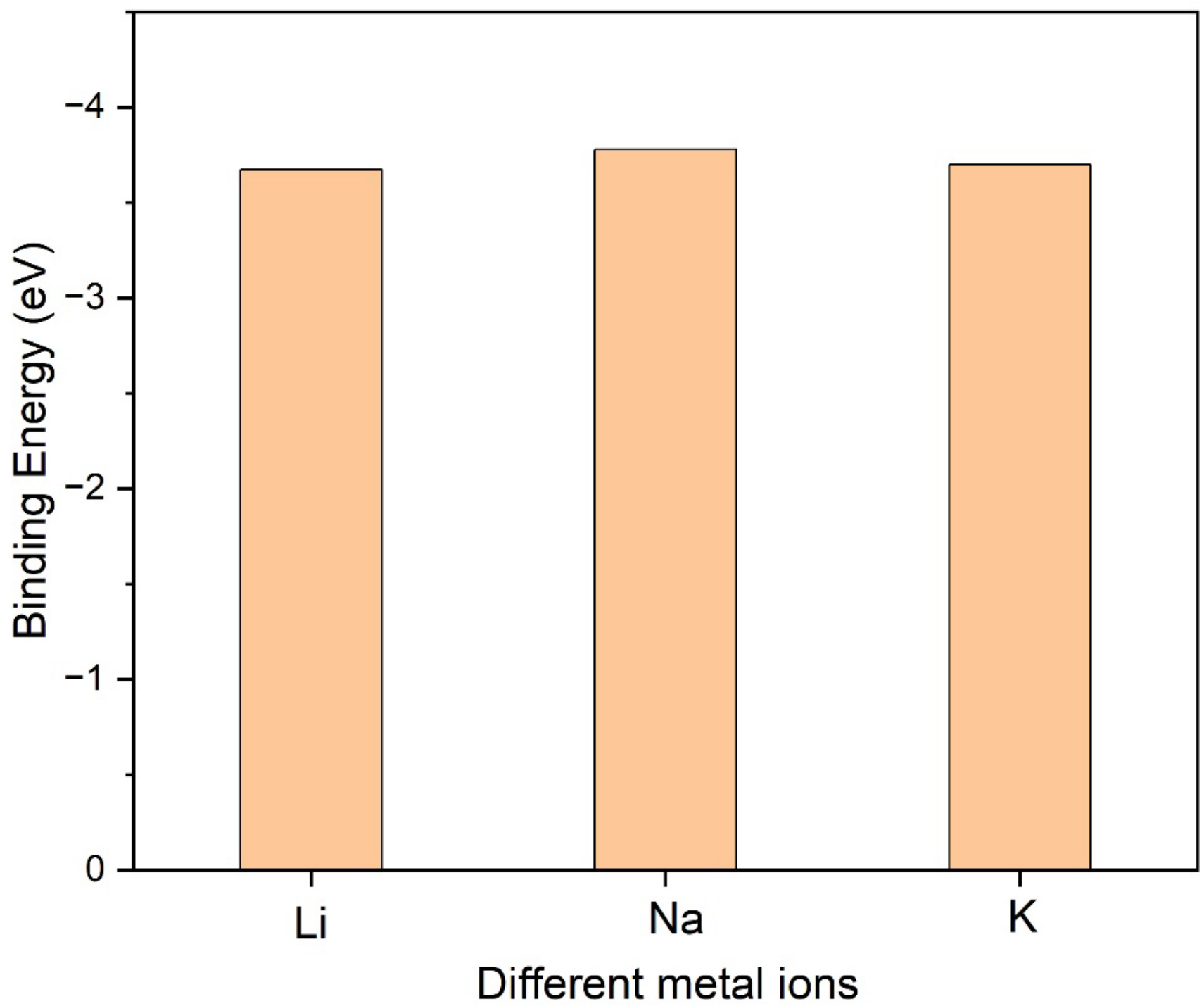

Figure S1. Calculated binding energies of Li^+, Na^+, and K^+ with $[Py_{14}][TFSI]$. The Li^+ and Na^+ values are identical to those presented in Figure 1d and are included here for direct comparison with the newly calculated K^+ value.

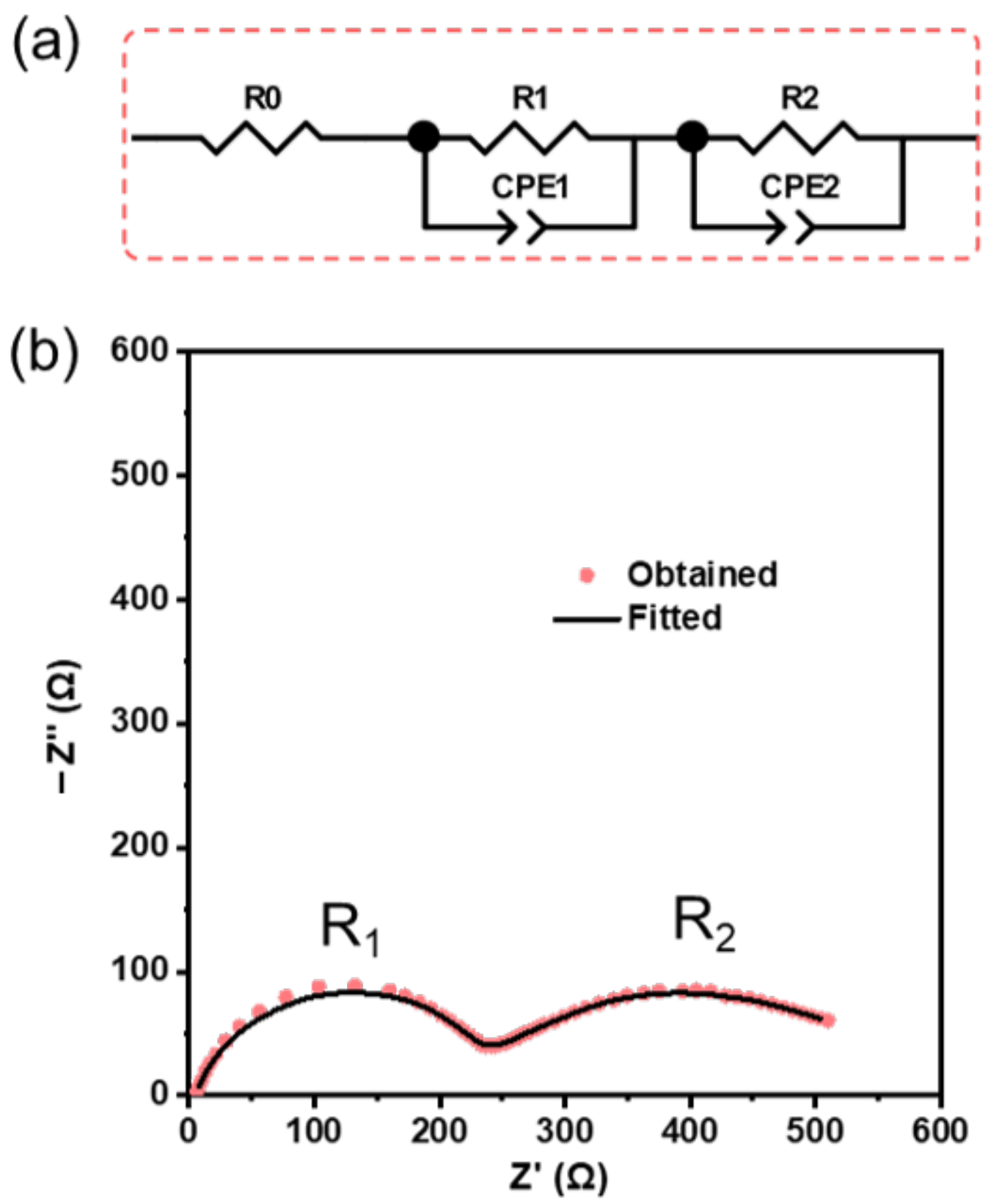

Figure S2. (a) Equivalent circuit model and (b) the corresponding semicircular regions for R1 and R2 in the fitted Nyquist plots of the symmetric cell at varying temperatures.

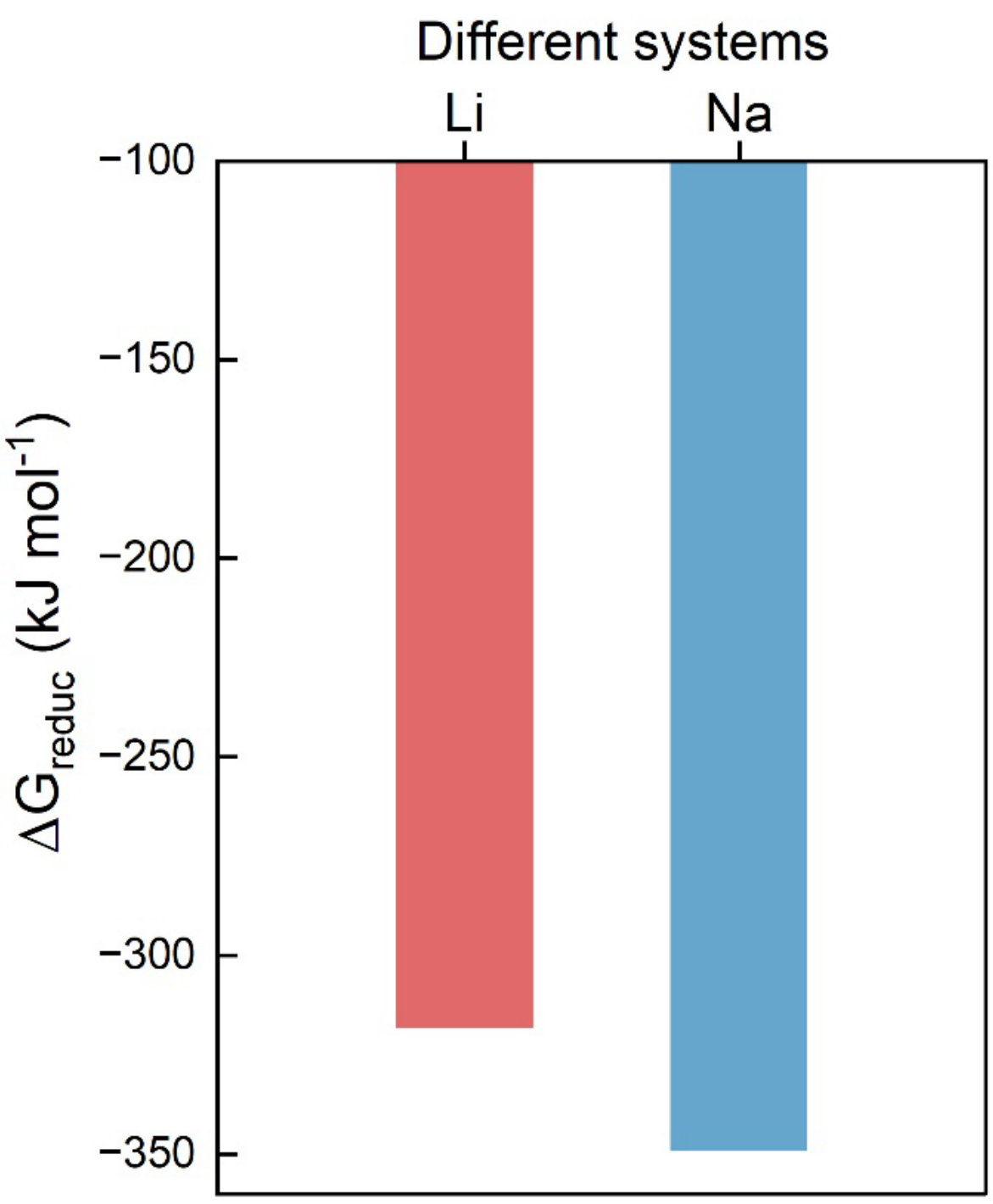

Figure S3. Deposition free energy of Li^+ and Na^+ in the G4 electrolyte.

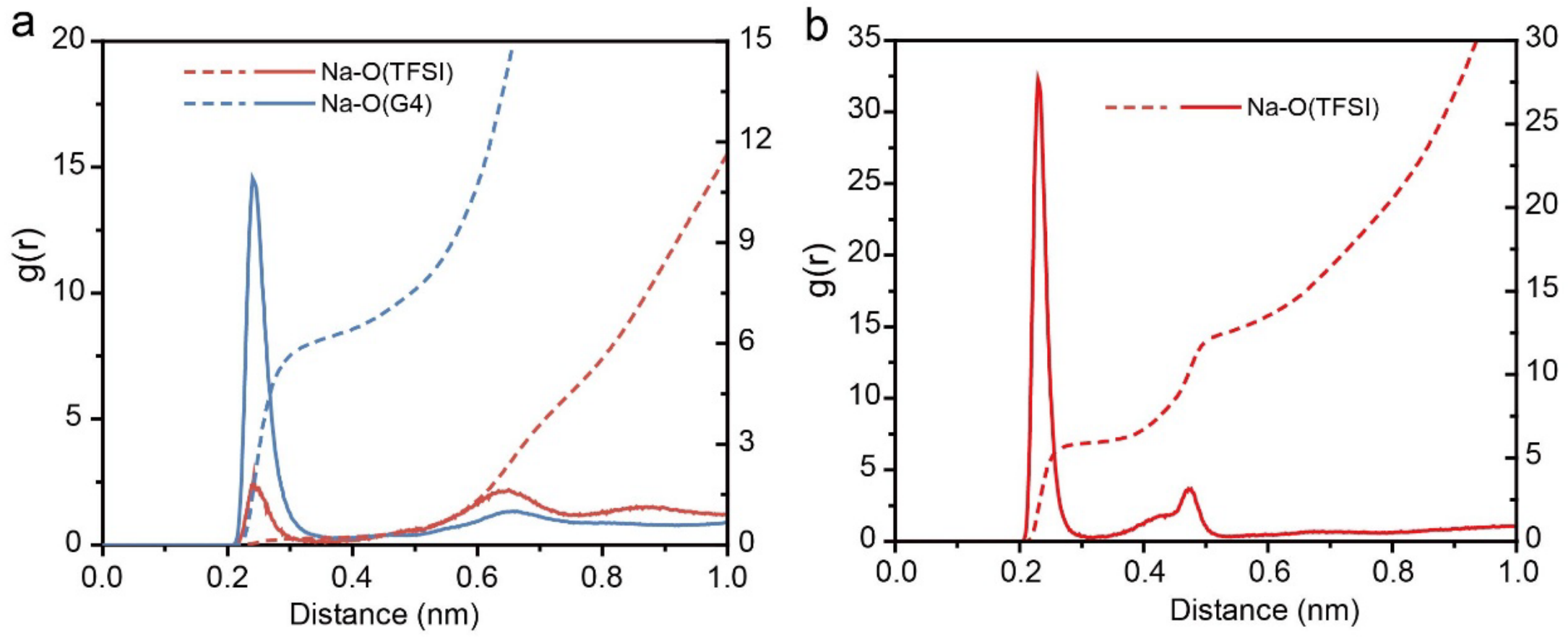

Figure S4. RDFs (solid lines) and coordination number (dashed lines) of (a) Na−O (TFSI, G4) for 3N-G4 and (b) Na−O (TFSI) for 3N-IL electrolyte.

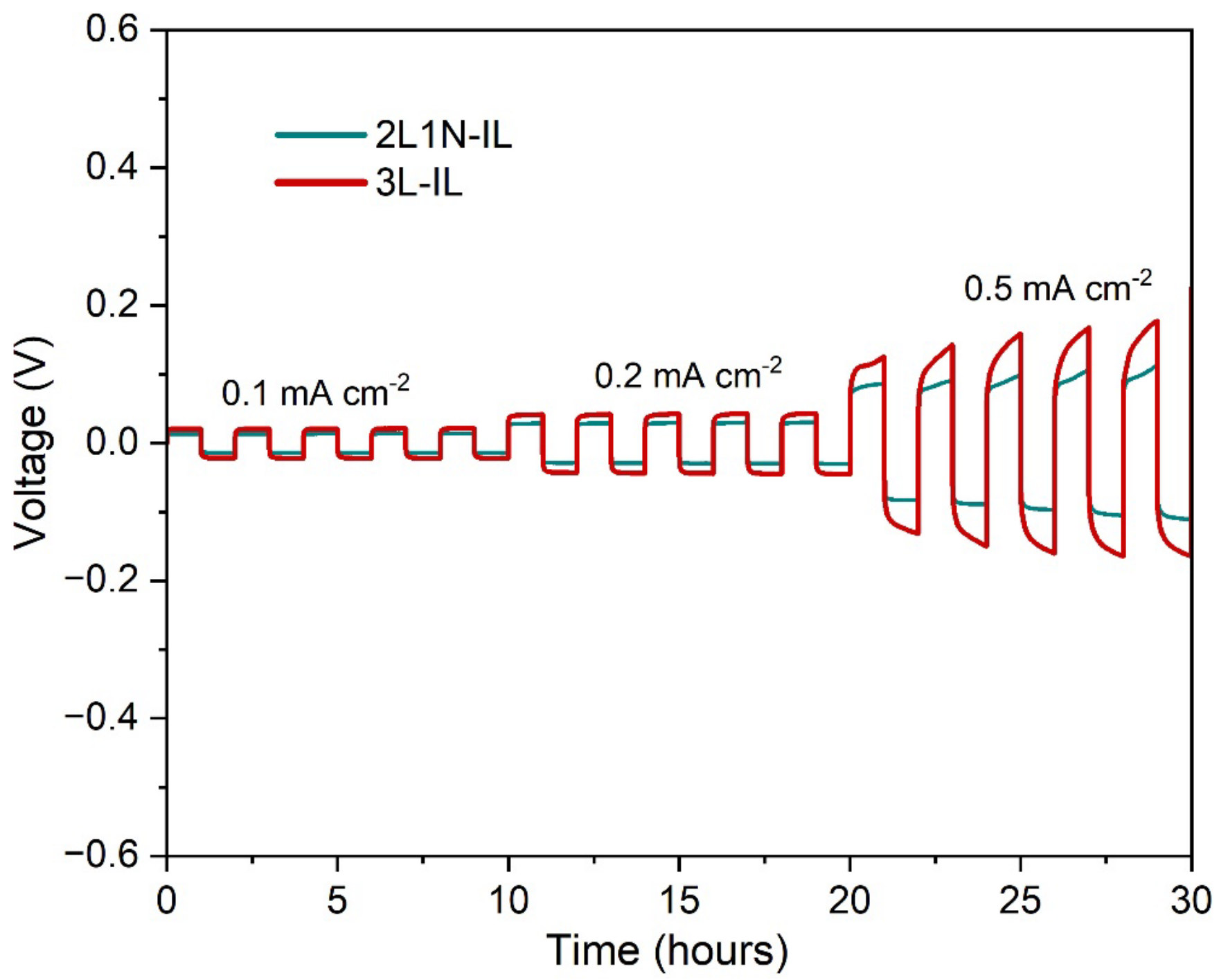

Figure S5. Galvanostatic voltage profiles of Li||Li symmetric cells using 3L-IL and 2L1N-IL electrolytes at sequentially increasing current densities of 0.1, 0.2, and 0.5 mA cm^{-2}.

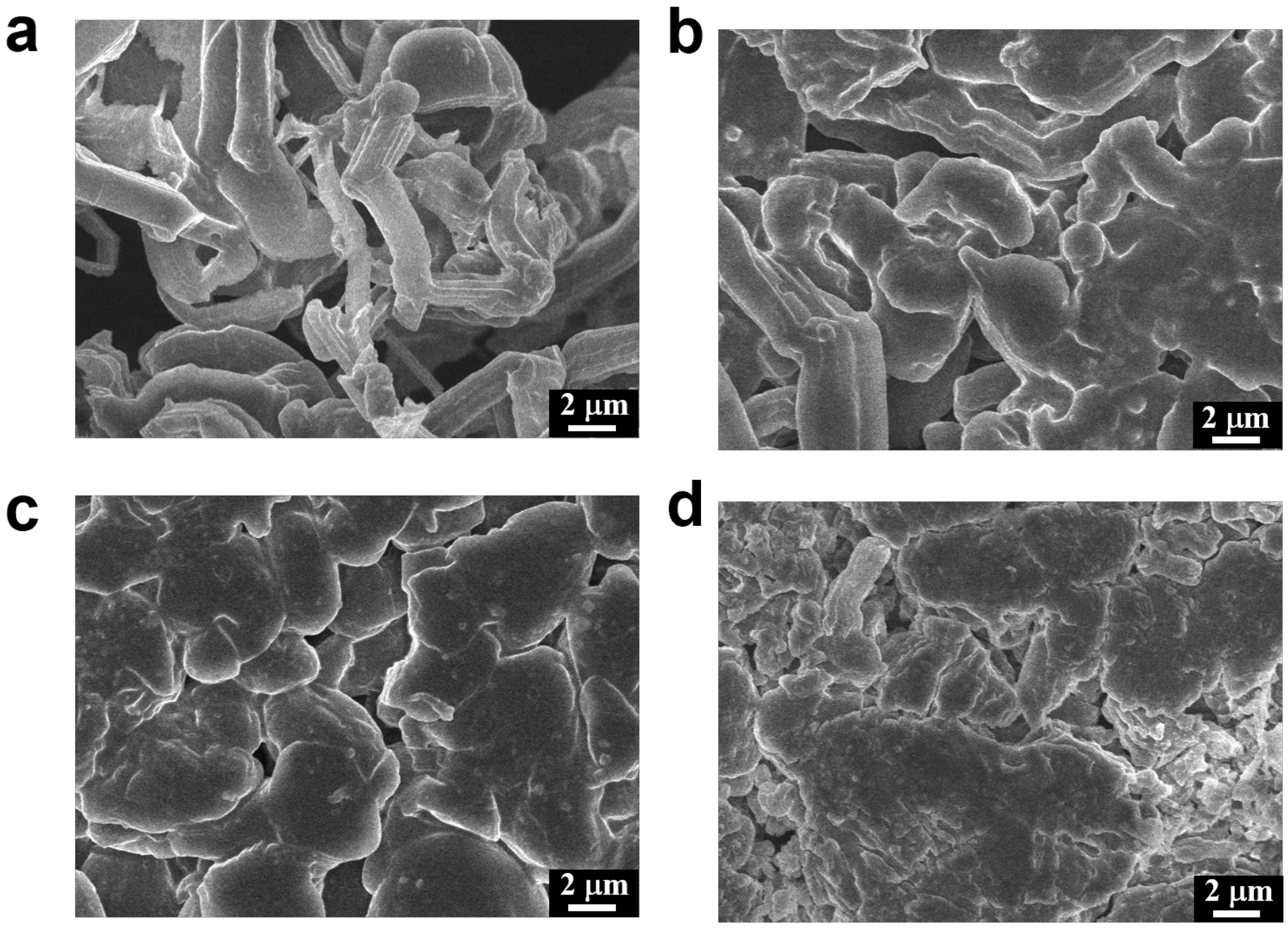

Figure S6. Enlarged views of the lithium metal electrode surface morphologies shown in Figure 5 after Li deposition to an area capacity of 5 mAh cm^{-2} at a current density of 0.2 mA cm^{-2} in different electrolytes: (a) 3L-IL, (b) 5L1N-IL, (c) 3L1N-IL, and (d) 1L1N-IL.

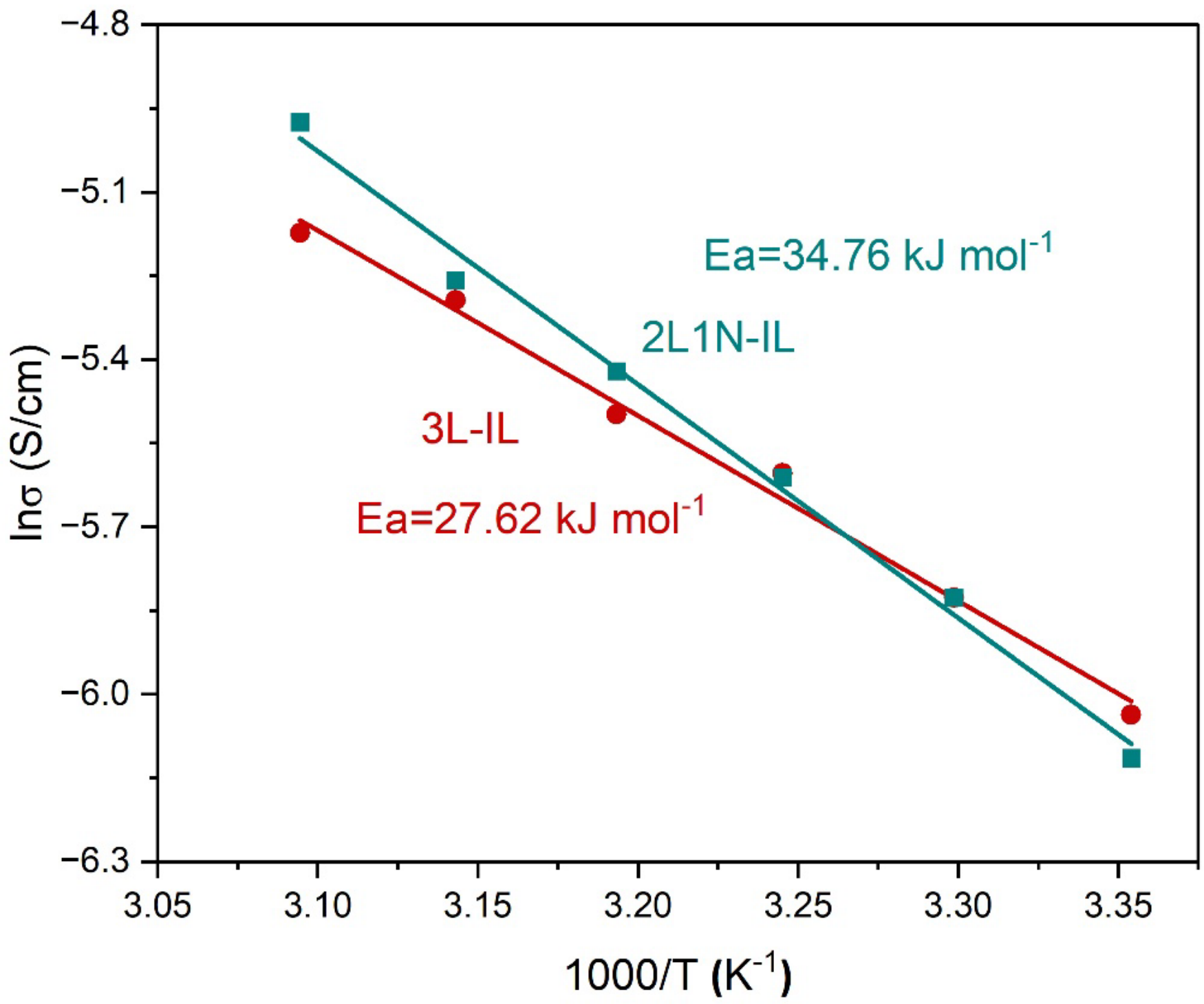

Figure S7. Arrhenius plots of ionic conductivity for 2L1N-IL and 3L-IL electrolytes over the temperature range of 25–50 °C.

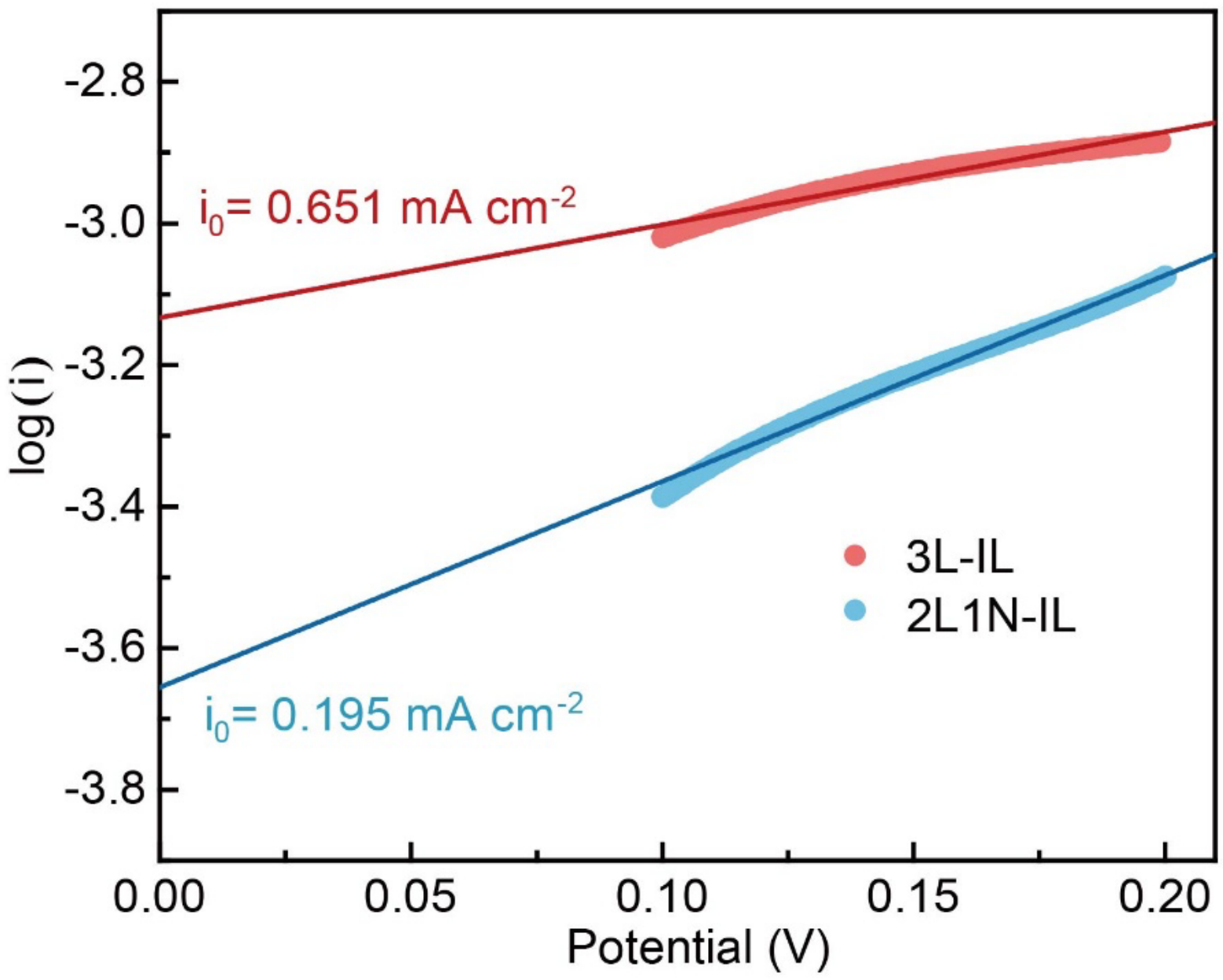

Figure S8. Comparison of the exchange current densities for lithium deposition/dissolution in the 3L-IL and 2L1N-IL electrolytes.

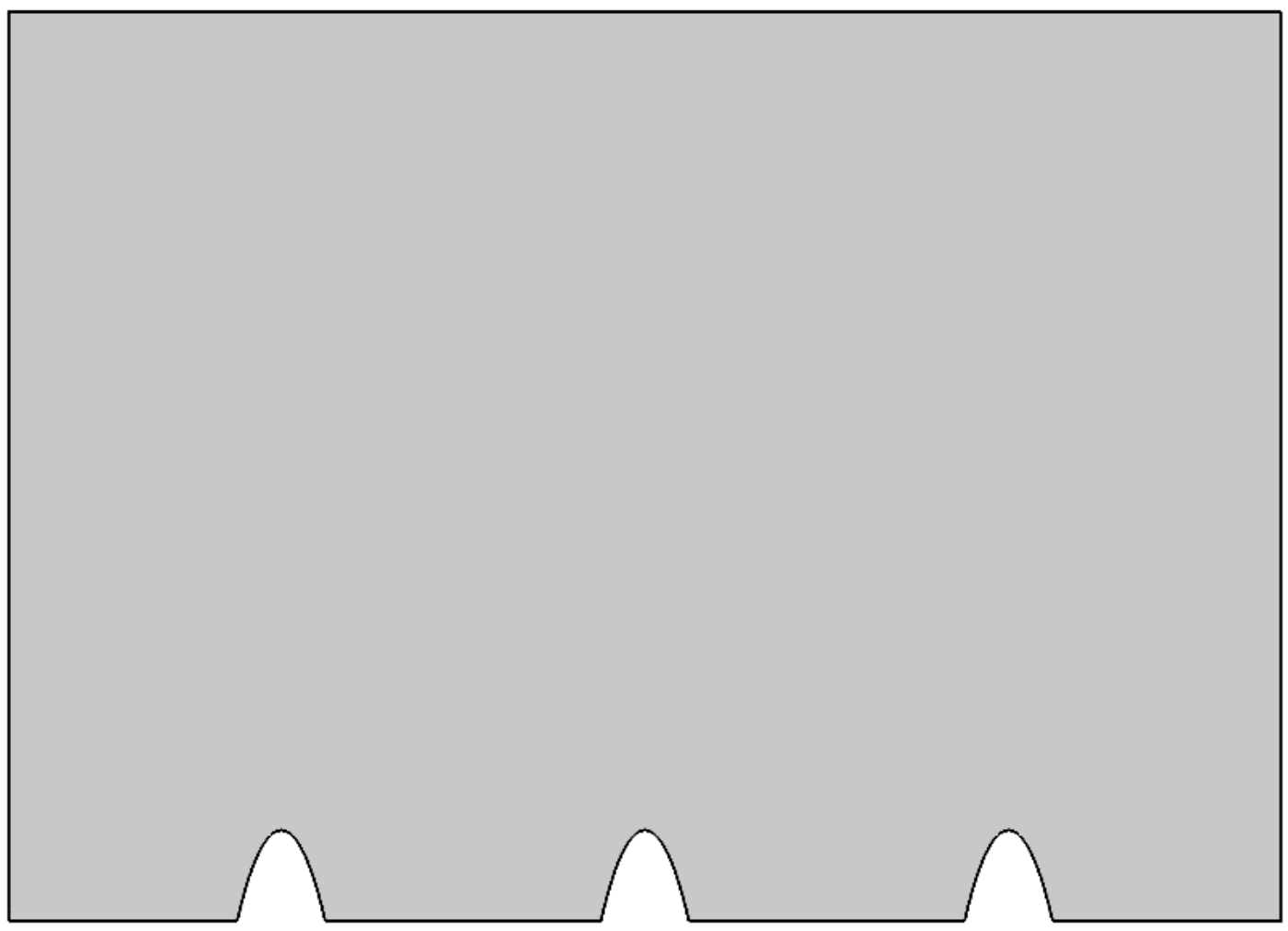

Figure S9. The pristine structure of cell constructed for Li deposition simulation

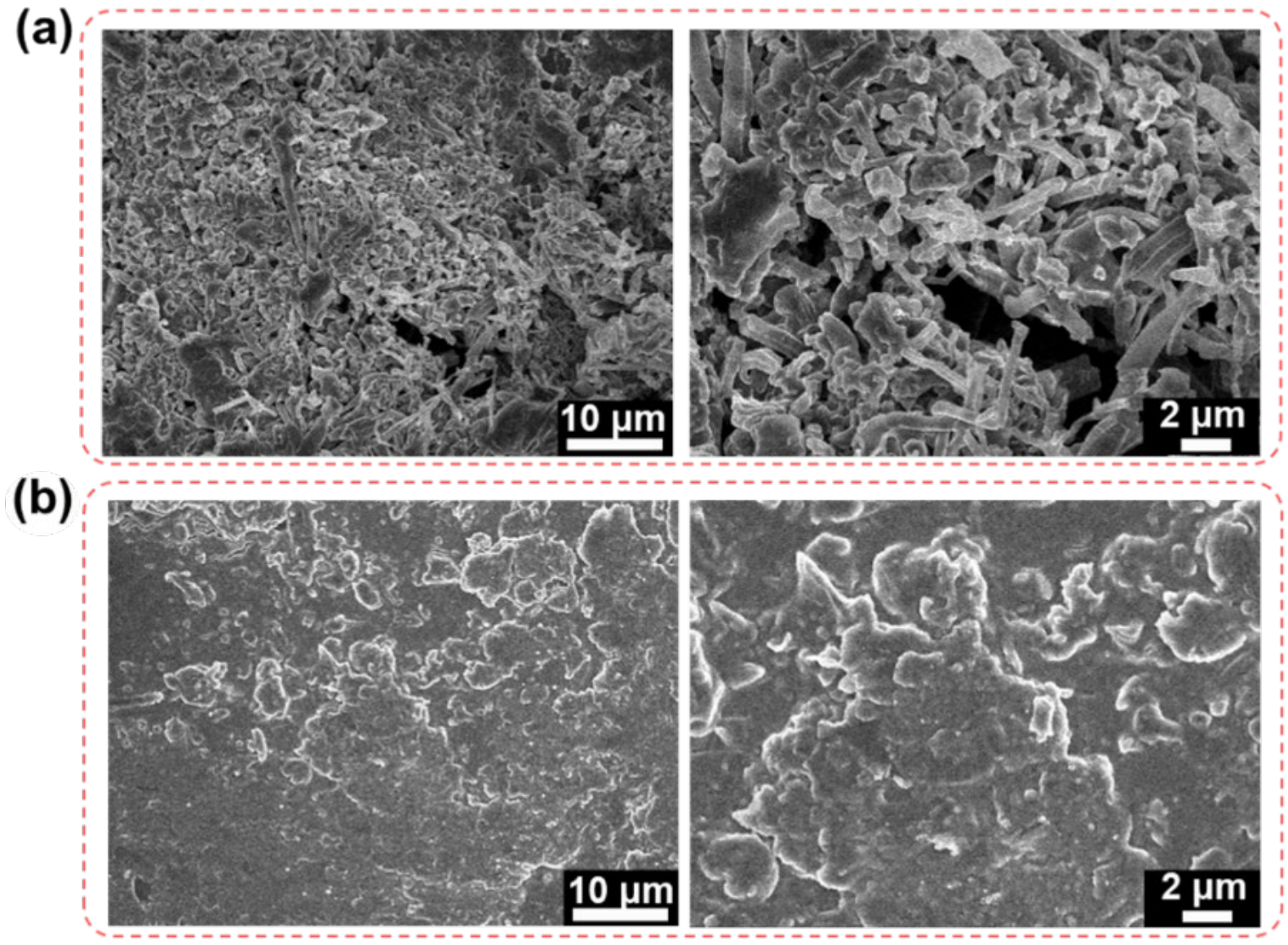

Figure S10. Surface morphologies of the lithium electrodes from Li||Li symmetric cells after 10 cycles using (a) the 3L-IL electrolyte and (b) the 2L1N-IL electrolyte.

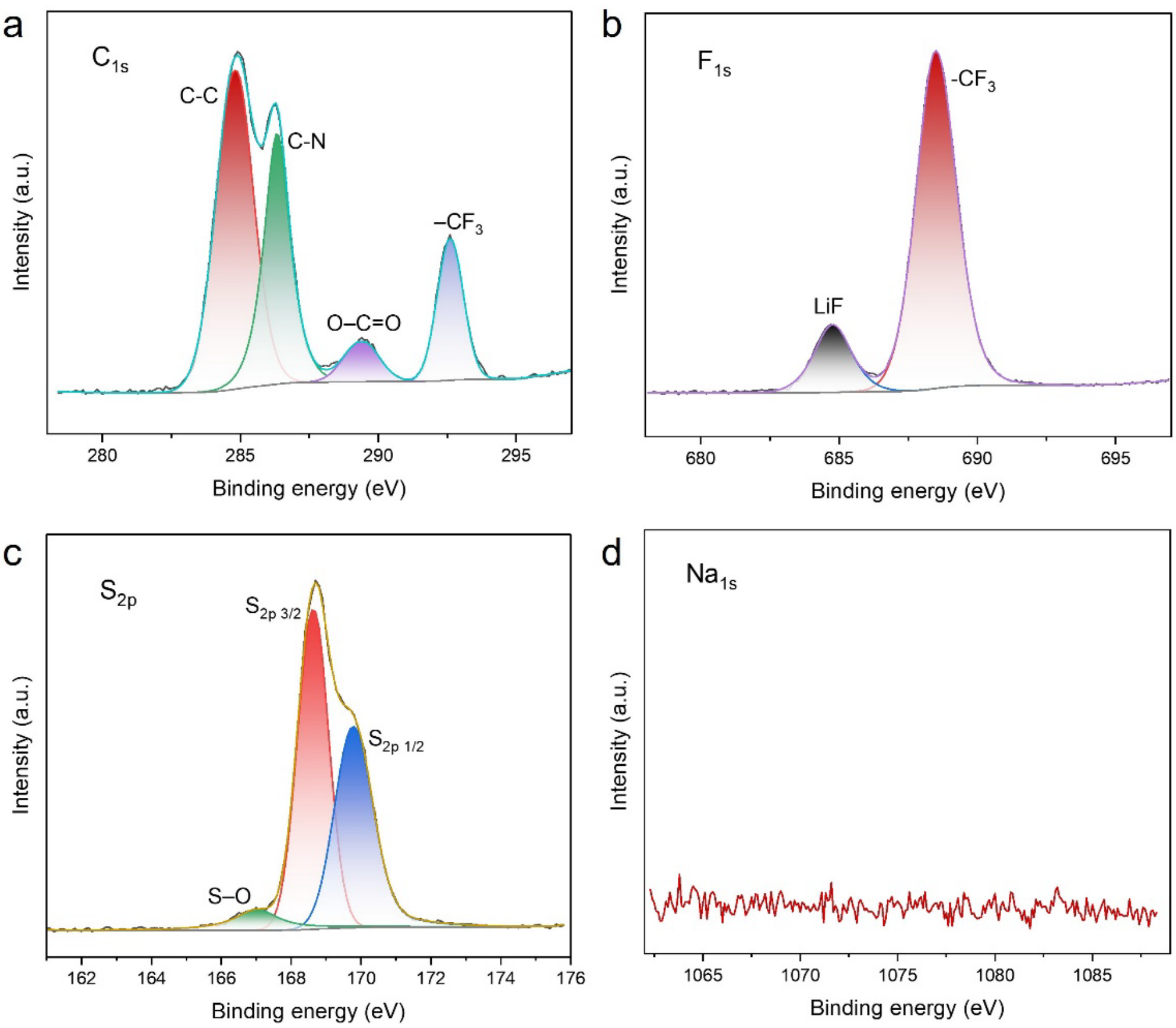

Figure S11. XPS spectra of a Li electrode recovered from a Li||Li symmetric cell after 20 cycles in 2L1N-IL: (a) C 1s, (b) F 1s, (c) S 2p, and (d) Na 1s.

Table S2. Comparison of lithium anode protection performance using ionic liquids in Li–O_2 batteries.

Ionic Liquid	Li‖Li symmetric cells Current density (mA cm^{-2})	Li‖Li symmetric cells Cycling time (h)	Li–O_2 cells Current density	Li–O_2 cells Limited Capacity	Li–O_2 cells Cycling life (cycles)	Ref.
1,2-dimethyl-3-(4-(2,2,6,6-tetramethyl-1-oxyl-4 piperidoxyl) pentyl)imidazolium bis(trifluoromethane)sulfonimide (IL-TEMPO)	1	600	0.1 mA cm^{-2}	0.25 mAh cm^{-2}	200	[11]
Poly (1-Butyl-3-vinylimidazolium bis(trifluoromethanesulfonyl imine)) ([PBVIm]-TFSI)	0.25	560	200 mA g^{-1}	500 mAh g^{-1}	105	[12]
1-butyl-1-methylpyrrolidinium bis((trifluoromethyl)sulfonyl)imide ([$Pyrr_{14}$][TFSI])	0.05	900	60 mA g^{-1}	500 mAh g^{-1}	60	[13]
1,3-dimethylimidazolium iodide (DMI)	0.1	1000	500 mA g^{-1}	1000 mAh g^{-1}	240	[14]
1,3-dimethyl-2-imidazolidinone (DMI)	1	640	400 mA g^{-1}	1000 mAh g^{-1}	205	[15]
(N,N-diethyl-N-methyl-N-(2-methoxyethyl)ammonium bis(trifluoromethanesulfonyl)imide) [DEME][TFSI]	0.2	550	200 mA g^{-1}	500 mAh g^{-1}	75	[16]
[Py_{14}][TFSI]	0.2	1400	500 mA g^{-1}	500 mAh g^{-1}	124	This work

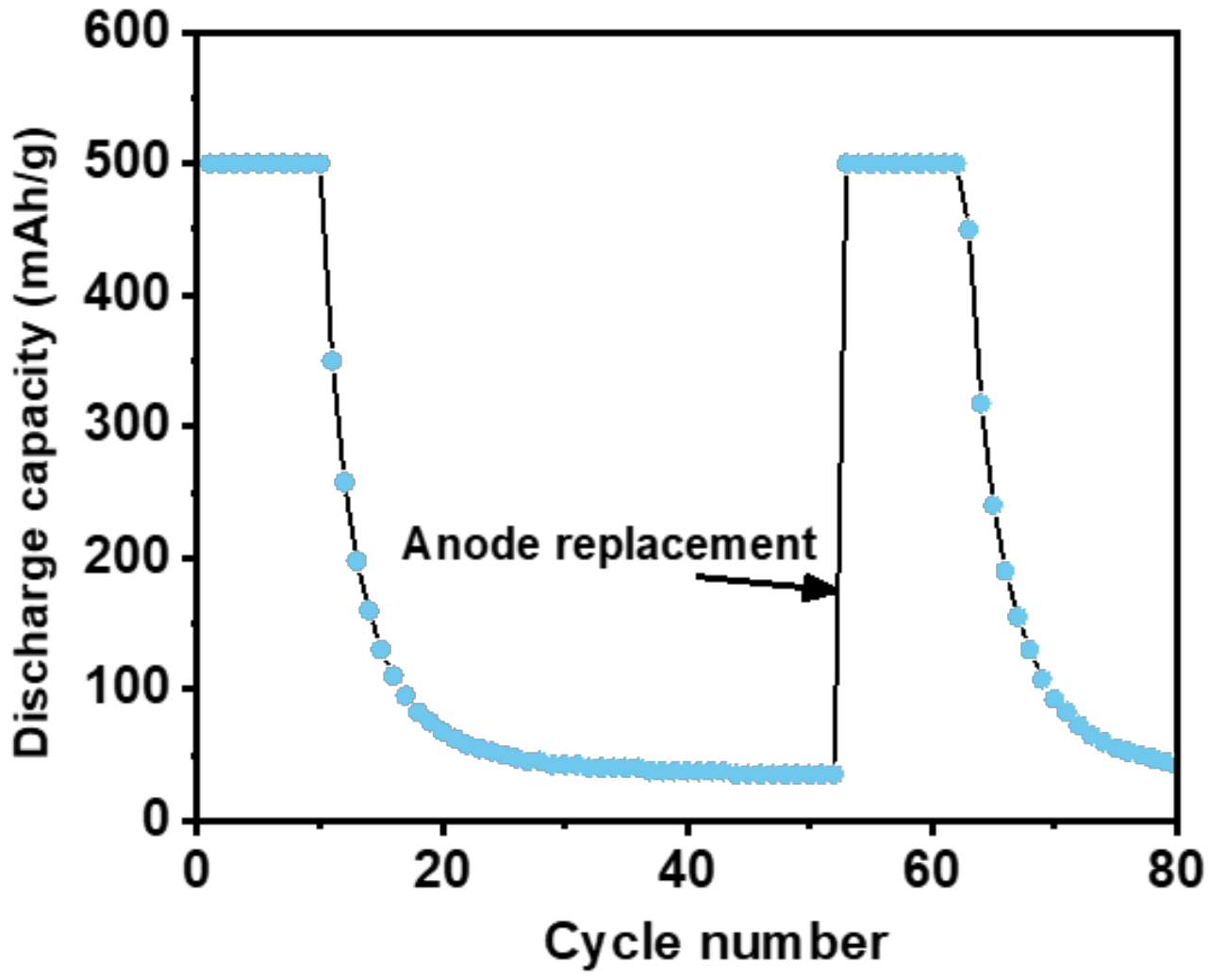

Figure S12. Discharge specific capacity of the Li-O_2 battery assembled with the 3L-IL electrolyte.

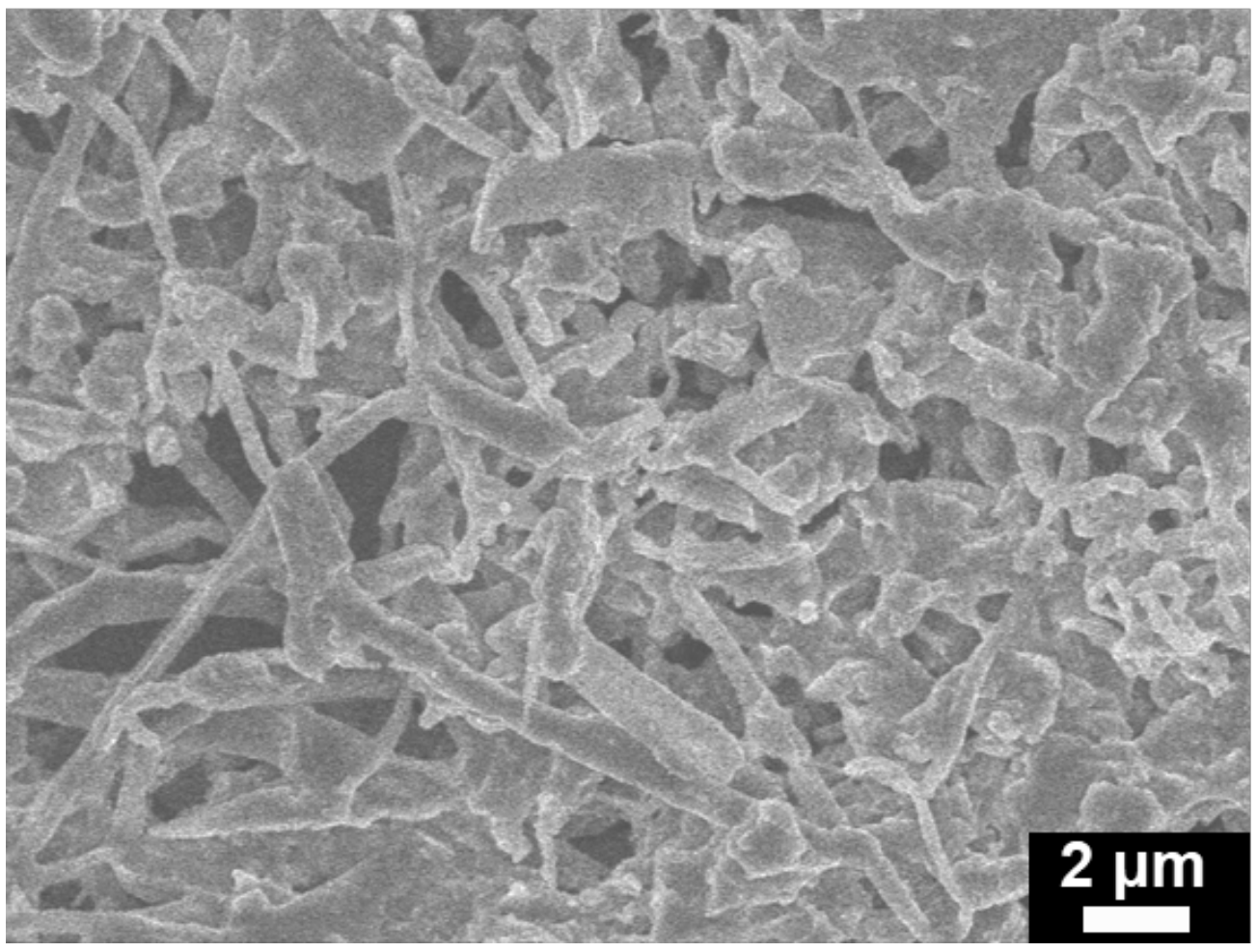

Figure S13. Surface morphology of the lithium anode from a failed Li-O_2 battery with the 3L-IL electrolyte.

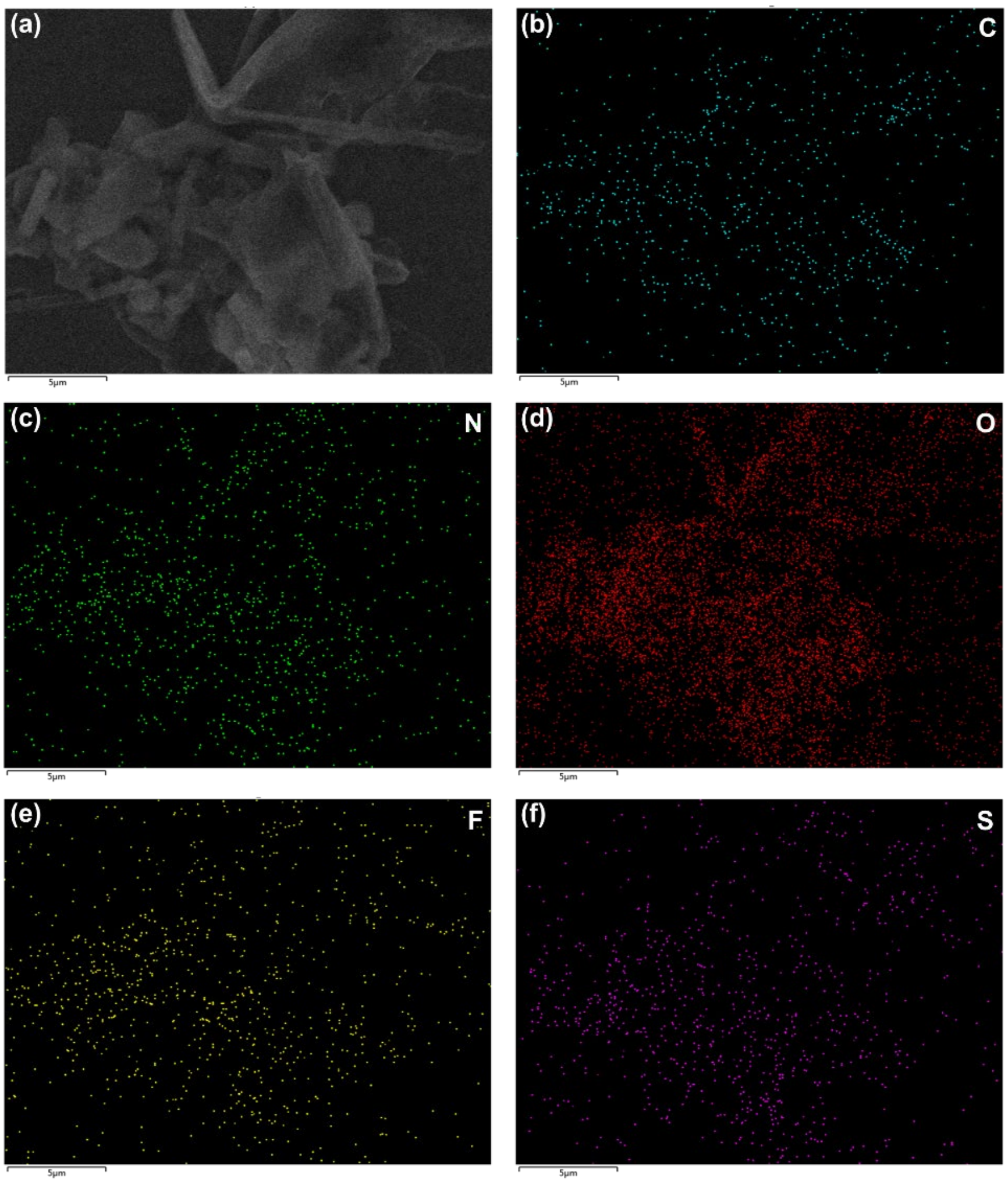

Figure S14. X-ray energy-dispersive spectroscopy (EDS) analysis of dendritic residues on the cycled lithium anode: (a) region of interest and (b-f) the corresponding EDS elemental mapping for (b) carbon, (c) nitrogen, (d) oxygen, (e) fluorine, and (f) sulfur.

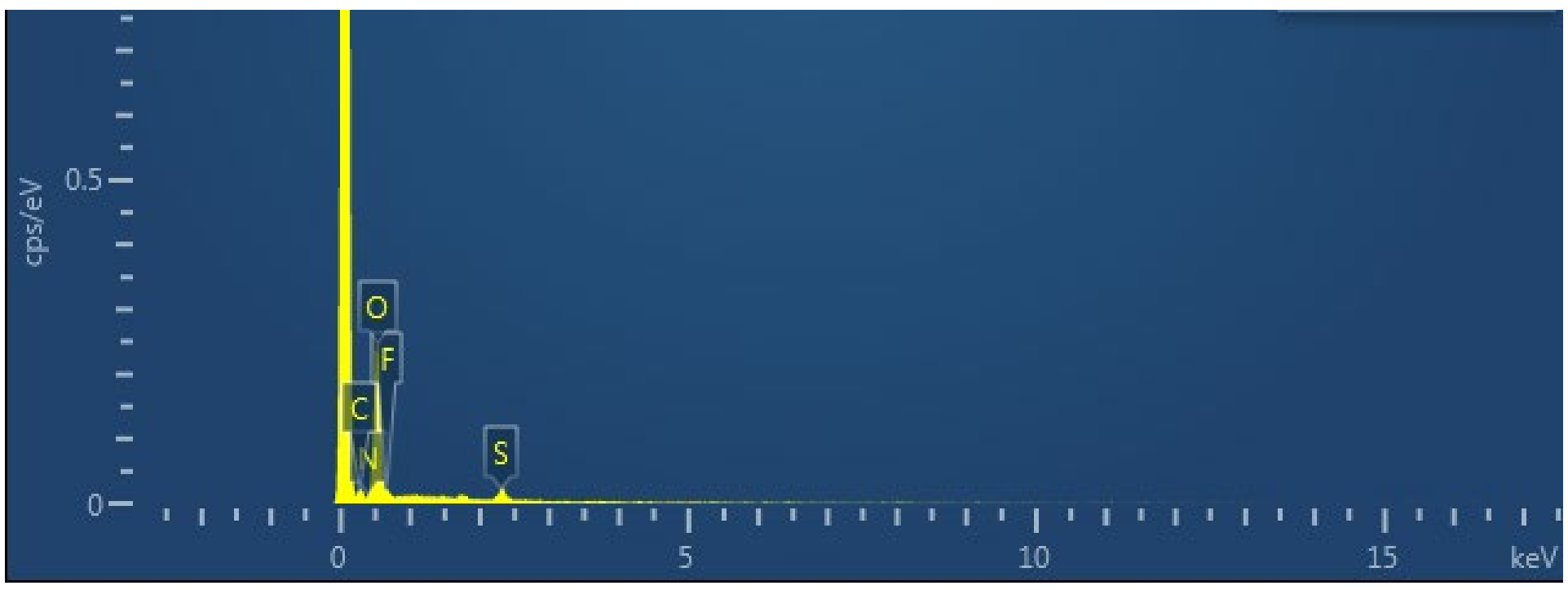

Figure S15. X-ray energy spectrum of dendritic residue on the lithium anode after cycling.

Table S3. EDS results of dendritic residue on the lithium anode after cycling.

Elements	Atomic percentage (at.%)
C	20.45
N	9.32
O	63.13
F	6.42
S	0.69

References

[1] Frisch, M.; Trucks, G.; Schlegel, H.; Scuseria, G.; Robb, M.; Cheeseman, J.; Scalmani, G.; Barone, V.; Petersson, G.; Nakatsuji, H.; et al. Gaussian 16, Revision A.03, Gaussian, Inc., Wallingford CT, **2016**.

[2] H.J. Berendsen, D. van der Spoel, R. van Drunen, GROMACS: A message-passing parallel molecular dynamics implementation *Comput. Phys. Commun.* **1995**, *91*, 43–56.

[3] M.J. Abraham, T. Murtola, R. Schulz, S. Páll, J.C. Smith, B. Hess, E. Lindahl, GROMACS: High performance molecular simulations through multi-level parallelism from laptops to supercomputers *Software X* **2015**, *1-2*, 19–25.

[4] J. Wang, R.M. Wolf, J.W. Caldwell, P.A. Kollman, D.A. Case, Development and testing of a general amber force field *J. Comput. Chem.* **2004**, *25*, 1157–1174.

[5] J. Wang, W. Wang, P.A. Kollman, D.A. Case, Automatic atom type and bond type perception in molecular mechanical calculations *J. Mol. Graph. Model.* **2006**, *25*, 247–260.

[6] A.W. Sousa da Silva, W.F. Vranken, ACPYPE-AnteChamber PYthon Parser interfacE BMC *Res. Notes* **2012**, *5*, 367.

[7] U.C. Singh, P.A. Kollman, An approach to computing electrostatic charges for molecules *J. Comput. Chem.* **1984**, *5*, 129–145.

[8] B.H. Besler, K.M. Merz Jr., P.A. Kollman, Atomic charges derived from semiempirical methods *J. Comput. Chem.* **1990**, *11*, 431–439.

[9] A. Schäfer, H. Horn, R. Ahlrichs, Fully optimized contracted Gaussian basis sets for atoms Li to Kr *J. Chem. Phys.* **1992**, *97*, 2571–2577.

[10] W. Humphrey, A. Dalke, K. Schulten, VMD: visual molecular dynamics *J. Mol. Graph.*

1996, *14*, 33–38.

[11] J. Zhang, B. Sun, Y. Zhao, A. Tkacheva, Z. Liu, K. Yan, X. Guo, A.M. McDonagh, D. Shanmukaraj, C. Wang, T. Rojo, M. Armand, Z. Peng, G. Wang, A versatile functionalized ionic liquid to boost the solution-mediated performances of lithium-oxygen batteries *Nat. Commun.* **2019**, *10,* 602.

[12] D. Li, Q. Chen, R. Li, Y. Hou, Y. Liu, H. Xie, J. Liu, J. Zhu, Polymeric Ionic Liquid-Enabled In Situ Protection of Li Anodes for High-Performance Li-O_2 Batteries *ChemSusChem* **2025**, *18*, e202402102.

[13] A.R. Neale, R. Sharpe, S.R. Yeandel, C.H. Yen, K.V. Luzyanin, P. Goddard, E.A. Petrucco, L.J. Hardwick, Design Parameters for Ionic Liquid–Molecular Solvent Blend Electrolytes to Enable Stable Li Metal Cycling Within Li–O_2 Batteries *Adv. Funct. Mater.* **2021**, *31*, 2010627.

[14] J. Liu, Y. Li, Y. Ding, L. Wu, J. Qin, T. Chen, C. Meng, F. Zhou, X. Ma, Z.S. Wu, A Bifunctional Imidazolyl Iodide Mediator of Electrolyte Boosts Cathode Kinetics and Anode Stability Towards Low Overpotential and Long-Life Li-O_2 Batteries *Angew. Chem. Int. Ed.* **2025**, *64*, e202421107.

[15] Z. Huang, J. Meng, W. Zhang, Y. Shen, Y. Huang, 1,3-Dimethyl-2-imidazolidinone: an ideal electrolyte solvent for high-performance Li-O_2 battery with pretreated Li anode *Sci. Bull.* **2022**, *67*, 141–150.

[16] Y. Cai, Q. Zhang, Y. Lu, Z. Hao, Y. Ni, J. Chen, An Ionic Liquid Electrolyte with Enhanced Li^+ Transport Ability Enables Stable Li Deposition for High-Performance Li-O_2 Batteries *Angew. Chem. Int. Ed.* **2021**, *60*, 25973–25980.
